# Guidance for Canadian Breast Cancer Practice: National Consensus Recommendations for the Systemic Treatment of Patients with HR+/HER2− Metastatic Breast Cancer 2025

**DOI:** 10.3390/curroncol33020106

**Published:** 2026-02-09

**Authors:** Katarzyna J. Jerzak, Aalok Kumar, Jean-François Boileau, Nathaniel Bouganim, Christine Brezden-Masley, Jeffrey Q. Cao, David W. Cescon, Stephen Chia, Scott Edwards, Anil Abraham Joy, Kara Laing, Nathalie LeVasseur, Sasha Lupichuk, Sandeep Sehdev, Christine Simmons, Marc Webster, Karen A. Gelmon, Mita Manna, on behalf of patient advocacy, Breast Cancer Canada

**Affiliations:** 1 Sunnybrook Odette Cancer Centre, University of Toronto, Toronto, ON M4N 3M5, Canada; katarzyna.jerzak@sunnybrook.ca; 2 BC Cancer— Surrey, University of British Columbia, Vancouver, BC V3V 1Z2, Canada; akumar7@bccancer.bc.ca; 3 Jewish General Hospital, McGill University, Montreal, QC H3T 1E2, Canada; jean-francois.boileau@mcgill.ca; 4 McGill University Health Centre, McGill University, Montréal, QC H4A 3J1, Canada; nathaniel.bouganim@mcgill.ca; 5 Marvelle Koffler Breast Centre, Mount Sinai Hospital, University of Toronto, Toronto, ON M5G 1X5, Canada; christine.brezden@sinaihealth.ca; 6 Arthur J.E. Child Comprehensive Cancer Centre, University of Calgary, Calgary, AB T2N 4N1, Canada; jeffrey.cao@albertahealthservices.ca (J.Q.C.); sasha.lupichuk@albertahealthservices.ca (S.L.); marc.webster@albertahealthservices.ca (M.W.); 7 Princess Margaret Cancer Centre, University of Toronto, Toronto, ON M5G 2M9, Canada; dave.cescon@uhn.ca; 8 BC Cancer—Vancouver, University of British Columbia, Vancouver, BC V5Z 4E6, Canada; schia@bccancer.bc.ca (S.C.); nathalie.levasseur@bccancer.bc.ca (N.L.); christine.simmons@bccancer.bc.ca (C.S.); 9 Dr. H. Bliss Murphy Cancer Center, Memorial University of Newfoundland, St. John’s, NL A1B 3V6, Canada; scott.edwards@nlhealthservices.ca (S.E.); kara.laing@nlhealthservices.ca (K.L.); 10 Cross Cancer Institute, University of Alberta, Edmonton, AB T6G 1Z2, Canada; anil.joy@albertahealthservices.ca; 11 The Ottawa Hospital Cancer Centre, Ottawa, ON K1H 8L6, Canada; ssehdev@toh.ca; 12 Faculty of Medicine, University of British Columbia, Vancouver, BC V5Z 1M9, Canada; kgelmon@gmail.com; 13 Saskatoon Cancer Centre, University of Saskatchewan, Saskatoon, SK S7N 4H4, Canada

**Keywords:** HR+ HER2− metastatic, advanced, breast cancer, Canadian consensus recommendations, evidence-based, REAL Alliance

## Abstract

Hormone receptor positive (HR+), human epidermal growth factor receptor 2-negative (HER2–) breast cancer accounts for approximately two-thirds of all breast cancers. The treatment landscape for HR+/HER2− metastatic breast cancer has evolved in recent years, with multiple new therapies demonstrating clinical efficacy and improved patient outcomes. To provide evidence-informed guidance on best practices in the management of patients with HR+/HER2− metastatic breast cancer in Canada, consensus recommendations were developed by Research Excellence, Active Leadership Canadian Breast Cancer Alliance (REAL Alliance), a standing nucleus committee with both clinical–academic oncologists from across Canada and representatives from Breast Cancer Canada, a patient advocacy organization. Recommendations were generated using a modified Delphi approach involving up to three anonymous voting rounds, with a predefined consensus threshold of 75%. These consensus recommendations offer guidance on optimal therapeutic strategies for HR+/HER2− metastatic breast cancer within the Canadian landscape, including integration of precision oncology and targeted therapies, optimal sequencing of therapies, and the importance of balancing the benefits of treatments with patient quality of life and preferences.

## Simple Summary

Hormone receptor positive (HR+) breast cancer is the most common type of breast cancer. In this subtype of breast cancer, growth is promoted by hormones, like estrogen and progesterone. Although HR+ cancers may also be human epidermal growth factor receptor 2 (HER2)-positive, this manuscript is restricted to HR+ HER2-negative (HER2–) cancers. The main initial treatment for HR+/HER2− breast cancer is endocrine therapy. In the last few years, studies have shown that new targeted therapies, which may be combined with endocrine therapy, provide a greater effect in reducing cancer burden and slowing disease progression. Chemotherapy and antibody–drug conjugates are also used to treat patients with HR+/HER2− breast cancer, typically after the cancer becomes resistant to endocrine-based therapy. In this publication, REAL Canadian Breast Cancer Alliance provides guidance to help healthcare providers, in collaboration with their patients, determine which patients may benefit from these treatments and the optimal order in which to offer them.

## 1. Introduction

Breast cancer is the most common cancer diagnosed in Canadian women and the second most common cancer diagnosed among all Canadians [1]. Epidemiologic data show that 64–76% of breast cancers are hormone receptor positive (HR+) and human epidermal growth factor 2 negative (HER2–) [2,3,4,5].

The goal of systemic treatment for metastatic HR+/HER2− breast cancer is to support patients’ wellbeing by stabilizing their disease and/or reducing disease burden, delaying progression, improving quality of life, and prolonging overall survival (OS) as metastatic disease is generally not curable [6,7,8,9].

Major therapeutic advances in recent years have dramatically improved clinical outcomes, including both duration and quality of life, for patients with HR+/HER2− metastatic breast cancer who previously had limited treatment options [10]. With the rapidly evolving treatment landscape and newer targeted therapies, managing patients has now shifted away from a “one-size-fits-all” model to an approach that considers individualized therapy, often aided by genomic testing.

The equitable standing nucleus committee—Research Excellence, Active Leadership Canadian Breast Cancer Alliance (REAL Alliance)—comprises a group of disciplinary experts in breast cancer from across Canada and representatives from Breast Cancer Canada, a patient advocacy organization. Formed in December 2023, it provides a national ecosystem of leadership to develop evidence-informed guidance and recommendations for equitable breast cancer care. REAL Alliance publishes national clinical consensus recommendations with an intention to promote timely health policy, funding, and consistent clinical adoption based on research evidence and clinical expertise to ensure optimal outcomes for breast cancer patients across all provinces and territories in Canada.

To address clinical challenges in the metastatic breast cancer setting, REAL Alliance developed consensus recommendations regarding optimal therapeutic strategies for HR+/HER2− metastatic breast cancer within the Canadian landscape, including integration of precision oncology and targeted therapies, optimal sequencing of therapies, and the importance of balancing the benefits of treatments with patient quality of life and preferences.

## 2. Materials and Methods

### 2.1. Consensus Recommendation Process

This work used methodology consistent with that of previous publications by REAL Alliance [11]. As an expert consensus guidance document, the intent was to identify key, practice-informing studies, rather than to conduct a formal systematic review. Evidence identification was expert-informed and targeted, focusing on publications relevant to the systemic treatment of HR+/HER2− metastatic breast cancer. Three REAL Alliance members (K.J.J., K.A.G., A.K.) formed the sub-committee that reviewed and identified the most relevant evidence. Based on this evidence, the sub-committee developed preliminary recommendations for systemic treatment of patients with HR+/HER2− metastatic breast cancer, each supported by pertinent references.

As stated in Manna et al. 2024 [11] and summarized here, these recommendations were evaluated using a modified Delphi process. Using an electronic platform, an initial round of anonymous voting was carried out by an expert panel. The panel comprised disciplinary experts from across Canada with expertise and extensive experience in managing breast cancer, including 14 medical oncologists, one surgical oncologist, one radiation oncologist, one oncology pharmacist, and one patient advocacy representative. Through an anonymous voting process, panelists independently rated their level of agreement with each statement, proposed modifications, and offered feedback on the cited literature and contextual evidence.

During a two-day, in-person consensus meeting in Toronto, ON, Canada, in April 2025, the supporting data were presented and the wording of the recommendations was discussed and revised as needed. Thereafter, a second round of electronic voting took place during which participants reviewed a summary of the discussion and the corresponding references, and anonymously evaluated their agreement with each recommendation. After completing a final round of edits, there was a third and final round of anonymous electronic voting. If 75% or more of the participants agreed with the statement after the predefined three rounds of revisions and voting, a recommendation was considered accepted. The consensus voting results are presented in Table S1.

The strength of recommendations was defined as follows: a strong recommendation is based on the highest quality of evidence; a moderate recommendation is supported by evidence that is less robust, indirect, or limited in scope; a weak recommendation is based on lower quality evidence; expert opinion reflects recommendations where there is endorsement by REAL Alliance but for which evidence is limited. The assessment of evidence quality was informed by expert interpretation of study design, relevance of the comparator, and robustness of the available data; a formal evidence grading system was not applied. As outlined in the recommendation tables in this guidance, the sub-committee compared REAL consensus recommendation alignment with the current European Society For Medical Oncology (ESMO) and American Society of Clinical Oncology (ASCO) guidelines.

### 2.2. Guiding Principles

The guiding principles were outlined in previous REAL Alliance publications [11] and are restated and revised here. At the outset of the development of these recommendations, REAL Alliance agreed that it is essential to consider the most effective therapies for both early and metastatic disease without consideration of access or reimbursement constraints which change over time.

Recommendations herein are arranged according to the line of therapy for metastatic HR+/HER2− breast cancer; however, treatment decisions should consider both disease biology and patient-specific factors (e.g., disease burden, symptoms, comorbid conditions, functional status, preferences, and values). Clinical decisions should remain individualized rather than dictated by rigid, predefined sequencing restrictions. Incorporation of patient preferences and values is essential. Shared decision-making should be broadly employed to ensure that patients understand the risks/benefits of proposed therapies and the relevant monitoring requirements, which must align with their treatment goals and preferences. The ESMO Magnitude of Clinical Benefit Scale (ESMO-MCBS) version 2.0 score, which indicates the magnitude of clinical benefit for recommended therapies, is presented where applicable (https://www.esmo.org/guidelines/esmo-mcbs/esmo-mcbs-for-solid-tumours/esmo-mcbs-scorecards, accessed on 13 January 2026).

Whenever aromatase inhibitors (AIs) are discussed, it is mandatory that pre and perimenopausal women receive the treatment alongside ovarian function suppression (OFS) and men receive a luteinizing hormone-releasing hormone (LHRH) agonist to ensure efficacy [12]. Options for chemical OFS in Canada include leuprolide and goserelin, which are LHRH agonists effective in reducing ovarian estrogen in premenopausal women with HR+ breast cancer [13].

The term “patient” is the used throughout the consensus recommendations to refer to ciswomen and cismen with HR+/HER2− metastatic breast cancer. Recommendations for transgender patients must consider their exogenous endocrine maintenance therapy and ideally would be managed with multidisciplinary care including an endocrinologist.

Though the statements are written to be as clear as possible, the reader should always read the accompanying narrative for the supporting evidence to fully appreciate the nuance associated with any given statement.

As stated in Manna et al. 2024 [11], enrolment in clinical trials is always encouraged for patients who meet the clinical trial eligibility criteria.

## 3. Systemic Therapy in HR+/HER2− Metastatic Breast Cancer

Endocrine therapy (ET) is a central component of therapy for patients with HR+/HER2− breast cancer. In the metastatic setting, the addition of a cyclin-dependent kinase 4 and 6 inhibitor (CDK4/6i) to an ET backbone is the first-line standard-of-care therapy for most patients [7,9,14,15]. Most patients with metastatic HR+/HER2− breast cancer will eventually develop a loss of response or resistance to systemic therapy, leading to disease progression and eventual death. As most breast cancers exhibit a continuum of sensitivity to ET, treatment should be tailored accordingly. In this guidance, we use the terms endocrine-sensitive, endocrine-eligible, and endocrine-ineligible to guide treatment selection. These categories reflect endocrine responsiveness as a continuum rather than as discrete “primary” or “secondary” resistance classifications.

Patients with endocrine-sensitive breast cancer have either not received prior ET (e.g., in the setting of de novo metastatic disease) or recent ET or have had their disease recur more than 12 months after completing adjuvant ET. Endocrine-eligible patients have breast cancer that retains some responsiveness to ET, despite disease relapse on or within 12 months of completing adjuvant ET or disease progression on ET in the metastatic setting. Such patients are endocrine-eligible because breast cancers that are still partially sensitive to estrogen may benefit from changing the ET backbone and/or adding targeted therapy depending on the clinical scenario and mutational status of the cancer. ESMO classifies these patients as having secondary (or acquired) endocrine resistance [16]. The choice between pursuing additional ET or transitioning to chemotherapy or an antibody–drug conjugate (ADC) should be guided by disease burden (e.g., low-volume or bone-predominant vs. extensive visceral disease), patients’ symptom burden, and pace of disease progression. In general, patients with symptomatic, rapidly progressive, and/or high-burden of visceral disease may be more appropriately managed with chemotherapy or ADCs as opposed to endocrine-based approaches. Patients who are endocrine-ineligible should not be treated with endocrine-based approaches due to extensive prior ET exposure with documented progression, suggesting endocrine-refractory biology.

**Figure 1:**
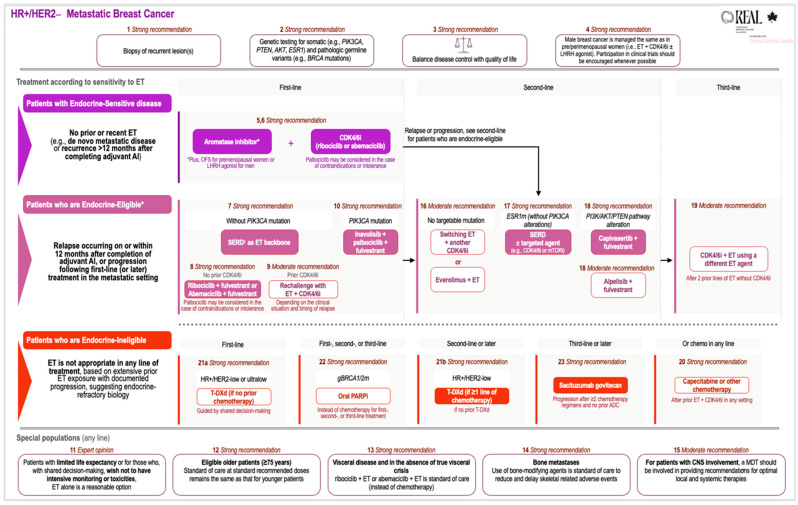
REAL Alliance recommendations for the management of HR+/HER2− metastatic breast cancer. Abbreviations: ADC = antibody–drug conjugates; CDK4/6i = cyclin-dependent kinase 4/6 inhibitor; CNS = central nervous system; *ESR1*m = *ESR1* mutation; ET = endocrine therapy; *gBRCA1/2m* = germline *BRCA1/2* mutation; LHRH = luteinizing hormone-releasing hormone; MDT = multidisciplinary team; mTORi = mammalian target of rapamycin inhibitor; OFS = ovarian function suppression; PARPi = poly-ADP ribose polymerase inhibitor. * Plus OFS for premenopausal women or LHRH agonist for men; ^†^ As of January 2026, fulvestrant is currently the only available SERD in this setting in Canada; newer generation oral SERDs should be considered as the ET backbone once approved by Health Canada.

REAL Alliance’s treatment recommendations are grouped into four broad categories covering line of therapy, timing of relapse, endocrine sensitivity, and special populations. Treatment recommendations for patients with HR+/HER2− metastatic breast cancer according to endocrine sensitivity are shown in Figure 1.

### 3.1. Preamble

Table 1 summarizes REAL Alliance recommendations for patients with HR+/HER2− metastatic breast cancer regarding biopsy, genomic testing, balancing disease control with quality of life, and breast cancer in men.

**Recommendation** **1.** *If feasible, biopsy of a recurrent lesion(s) should be considered at the time of diagnosis to evaluate biomarkers (ER/PR/HER2 status) and confirm a breast cancer diagnosis if the clinical course is not as expected. [Strong recommendation]*

Biopsy at disease recurrence or after an unclear response to therapy is crucial to confirm a diagnosis of breast cancer, reassess tumour biology (ER/PR/HER2 status), and when relevant, assess somatic pathway alterations to guide personalized therapy [9,14]. The current standard to evaluate tumour biology remains tissue biopsy. Although liquid biopsy for circulating tumour DNA (ctDNA) is a promising tool in precision oncology, it does not reliably determine tissue of origin or assess ER/PR/HER2 receptor status, which are routinely evaluated by immunohistochemistry in clinical practice.

Breast cancer is a complex, heterogeneous disease and tumour biology may vary between primary and metastatic tumours [20]. When there is discordance between primary and metastatic tumour biology, ASCO guidelines recommend management according to the metastatic tumour if the approach is clinically appropriate and aligned with the patient’s treatment goals [17]. Notably, primary or metastatic lesions can be used to assess HER2-low status and may render a patient eligible for trastuzumab deruxtecan (T-DXd), which is further discussed in Recommendation 21.

**Recommendation** **2.** *Testing for somatic pathway alterations (e.g., PIK3CA, PTEN, AKT, ESR1) and pathogenic germline variants, (e.g., BRCA mutations) is standard of care when it may inform treatment decisions. Testing should be performed with appropriate methods and in a timely manner to guide treatment decisions. [Strong recommendation]*

In line with international guidelines, REAL Alliance recommends assessing therapeutically relevant biomarkers as part of routine clinical practice to guide treatment decisions [14,21,22]. The clinical benefit of treatment with PI3K inhibitors in patients with *PIK3CA* somatic mutations was demonstrated in INAVO120 (first-line inavolisib + fulvestrant and palbociclib). For patients with PI3K/AKT/PTEN pathway alterations, CAPItello-291 demonstrated efficacy of capivasertib + fulvestrant. These trials are reviewed in Recommendations 10 and 18, respectively.

*ESR1* mutations are dynamic and become more common as the tumour progresses and therefore testing should be repeated at progression if negative at baseline. *ESR1* mutations are associated with resistance to AIs and can be detected in ctDNA preceding clinical progression [23,24]. Given subclonal and tumour heterogeneity, ctDNA is the standard method for detecting *ESR1* mutations [25], although polymerase chain reaction (PCR)-based assays may be considered for single-gene testing in certain clinical contexts. Early detection of *ESR1* mutations in ctDNA before radiographic progression may allow for treatment adjustments to delay clinical deterioration and improve outcomes for patients [23,26]. The results of the first-line SERENA-6 study in patients with emerging *ESR1* mutations are reviewed in Recommendation 17. As long-term outcomes, such as survival, have not yet been reported, more research and longer follow-up are needed prior to implementing routine ctDNA testing for *ESR1* mutations for patients with HR+/HER2− metastatic breast cancer.

**Recommendation** **3.** *In the management of HR+/HER2− metastatic breast cancer, it is essential to balance disease control with quality of life, allowing patients to maintain daily activities and minimize treatment-related discomfort. [Strong recommendation]*

REAL Alliance strongly suggests a multidimensional approach when managing patients with HR+/HER2− metastatic breast cancer considering the need for disease control to prevent and delay progression while balancing this with individual preferences and quality of life. While disease control is central in the management of patients, some persons will prefer one mode of delivery over another (oral versus injection), favour higher efficacy over mitigating side effects, fewer side effects over better efficacy, or prioritize quality of life over improved survival [27].

**Recommendation** **4.** *The treatment and management of breast cancer in men is the same as in pre/perimenopausal women (i.e., ET + CDK4/6i +/– LHRH agonist in the first-line metastatic setting). Given the rarity of breast cancer in men, participation in clinical trials should be encouraged whenever possible. [Strong recommendation]*

Breast cancer in men accounts for less than 1% of all breast cancer and historically has been underrepresented in clinical trials [28]. REAL Alliance strongly recommends men with metastatic breast cancer be considered for treatment in a clinical trial setting to further define optimal therapies for breast cancer in men. In men, hormone therapy options include tamoxifen, an AI in combination with a LHRH agonist, or selective estrogen receptor degraders (SERDs) [19]. Transgender persons require consideration for their exogenous hormones.

### 3.2. First-Line Metastatic Treatment for Patients with Endocrine-Sensitive Breast Cancer

Patients with endocrine-sensitive breast cancer are persons who have not received prior or recent ET, such as patients with de novo metastatic disease or those whose cancer recurred more than 12 months after completing adjuvant ET. Table 2 summarizes REAL Alliance recommendations for patients with endocrine-sensitive metastatic breast cancer.

**Recommendation** **5.** *For patients with HR+/HER2− metastatic breast cancer who have not received prior or recent ET, the standard of care ET backbone in the first-line metastatic setting is an AI. [Strong recommendation]*

An AI is the recommended ET backbone in patients with de novo metastatic breast cancer or disease that recurs >12 months after the completion of adjuvant ET [9,14,18].

There may be situations where tamoxifen is preferred instead of an AI, such as, when an individual cannot tolerate AIs because of side effects (e.g., severe arthralgia or osteoporosis) or in men with breast cancer who are unable to take an LHRH agonist. Tamoxifen carries its own risks, however, and it is not recommended in combination with ribociclib due to the compounded risk for QTc prolongation [29] and should be used with caution in patients receiving abemaciclib due to an elevated risk of venous thromboembolism [30].

**Recommendation** **6.** *For patients with de novo metastatic HR+/HER2− breast cancer, or those whose disease relapses >12 months after completing adjuvant AI, and regardless of adjuvant treatment with CDK4/6i, the standard of care first-line treatment is CDK4/6i + AI. The preferred CDK4/6i is either ribociclib or abemaciclib; however, in the case of contraindications or intolerance, palbociclib may be considered. [Strong recommendation]*

The phase 3 MONALEESA-7 study randomized 672 premenopausal women with HR+/HER2− advanced breast cancer to ribociclib + ET versus (vs) placebo + ET [31,32]. Notably, this is the only landmark trial designed exclusively for premenopausal women. The primary endpoint was met with a median progression-free survival (PFS) of 23.8 vs. 13.0 months with ribociclib + ET vs. placebo + ET, respectively [31]. At the final protocol-specified analysis, ribociclib + ET resulted in a 29% lower risk of death compared to ET alone, with median OS not reached for ribociclib + ET vs. 40.9 months with placebo + ET [32]. Health-related quality of life (HRQoL) was maintained longer with ribociclib and clinically meaningful deterioration in global health status was delayed by 1 year (median time to deterioration ≥10% was 35.8 vs. 23.3 months, respectively) [33]. With longer follow-up, ribociclib + ET continued to demonstrate a survival benefit, and no new safety signals were observed [34]. Grade 3/4 adverse events (AEs) of special interest were neutropenia (65%), hepatobiliary toxicity (12%), and prolonged QT interval (2%) [34]. For patients on ribociclib, QTc monitoring is required at baseline, on day 14 of cycle 1, and then as clinically indicated [29]. In addition, ribociclib is contraindicated in patients with untreated congenital long QT syndrome, a QTcF interval of ≥450 msec at baseline, and those who are at significant risk of developing QTc prolongation [ESMO-MCBS v2.0 score: 4].

MONALEESA-2 enrolled 668 postmenopausal women with HR+/HER- locally advanced or metastatic breast cancer who were randomized to first-line ribociclib + AI or placebo + AI. The primary endpoint of median PFS was not reached with ribociclib vs. 14.7 months with placebo (hazard ratio [HR] 0.56, 95% CI 0.43–0.72, *p* = 0.00000329) [35]. Overall, HRQoL was maintained throughout treatment [36]. At a median follow-up of 6.6 years, ribociclib + ET significantly extended median OS by more than 12 months (63.9 vs. 51.4 months; HR for death 0.76, 95% CI 0.63–0.93, *p* = 0.008) [37]. Grade 3 or 4 AEs included neutropenia (63.8%), hepatobiliary toxicity (14.4%), prolonged QT interval (4.5%), and interstitial lung disease (ILD; 0.6%) [ESMO-MCBS v2.0 score: 4].

MONARCH-3 evaluated first-line abemaciclib + ET (nonsteroidal AI [NSAI] with anastrozole or letrozole) vs. placebo + NSAI in 493 postmenopausal women with HR+/HER2− locally advanced or metastatic breast cancer. The study met its primary endpoint of PFS (median PFS 28.2 vs. 14.8 months; HR 0.54, 95% CI 0.41–0.72, *p* = 0.000021) [38]. The final OS analysis at a median follow-up of 8.1 years did not meet statistical significance, with a median OS of 66.8 months in the abemaciclib arm and 53.7 months in the placebo arm (HR 0.804, 95% CI 0.637–1.015, *p* = 0.0664), although the difference of 13.1 months is considered clinically important [39]. The most common grade 3 or 4 AEs were diarrhea (9.5%) and neutropenia (22.0%). Dose reductions (16.7%) and antidiarrheal medications (74.3%) were frequently used to manage diarrhea [38]. While no statistically significant detriment on HRQoL was observed on treatment, diarrhea was associated with a clinically meaningful negative impact on patients [40,41].

Although MONARCH-3 only enrolled a postmenopausal population, first-line abemaciclib + ET should be considered for pre/perimenopausal women and men given that these populations respond to therapy in a similar fashion as postmenopausal women [39,42] [ESMO-MCBS v2.0 score: 1].

Ribociclib demonstrated a statistically significant OS benefit, while abemaciclib offers continuous dosing and lack of drug interactions with antidepressants [43], medications that are frequently prescribed in people with breast cancer [44,45]. Abemaciclib, however, is associated with a high incidence of diarrhea, which can be distressing for patients, even if only grade 1, which is defined as an increase of less than 4 stools per day [46]. Dose modifications and anti-diarrheal medications can often alleviate this toxicity, and it may be mitigated initially by gradual abemaciclib dose escalation [47].

In cases where ribociclib and abemaciclib are unsuitable options due to contraindications or intolerance, palbociclib + AI may be considered as initial therapy based on a significant PFS benefit and safety observed in the phase 3 PALOMA-2 study [48,49,50]. PALOMA-2 enrolled 666 postmenopausal women with HR+/HER2− locally advanced or metastatic breast cancer without previous systemic therapy. After a median follow-up of 38 months, median PFS was 27.6 months with palbociclib + letrozole vs. 14.5 months with placebo + letrozole (*p* < 0.0001) [49]. No survival benefit was observed (median OS was 53.9 vs. 51.2 months, respectively; one-sided *p* = 0.34) [48]. The most common grade 3 AE associated with palbociclib was neutropenia [50] [ESMO-MCBS v2.0 score: 2].

The efficacy and safety outcomes of the phase 3 trials supporting the recommendations for treatment with CDK4/6i + AI in patients with endocrine-sensitive breast cancer in the first-line metastatic setting are presented in Table S2.

The survival benefit of first-line ET + CDK4/6i was demonstrated in the observational, prospective Treat ER + ight study, a real-world Canadian study on treatment patterns in HR+/HER2− advanced breast cancer. In this study, patients receiving ET + CDK4/6i in the first-line setting remained on treatment longer and had a significantly longer OS compared to patients receiving ET monotherapy (median OS: 25.9 months with ET vs. not estimable with ET + CDK4/6i; HR 0.30, *p* = 0.0024) [51].

### 3.3. First-Line Metastatic Treatment for Patients with Endocrine-Eligible Breast Cancer (Relapse on or ≤12 Months After Completion of Adjuvant AI)

In the first-line metastatic setting, endocrine-eligible persons include those recently treated with ET, whose disease remains partially responsive to ET but may demonstrate emerging resistance. In those patients whose disease has relapsed on or within 12 months after completion of adjuvant ET, testing for *PIK3CA* mutations should be undertaken [9,14].

Table 3 summarizes REAL Alliance recommendations for patients with endocrine-eligible metastatic breast cancer (relapse on or within 12 months after completion of adjuvant AI) according to *PIK3CA* status. Given a clinically meaningful and statistically significant PFS and OS benefit associated with inavolisib + palbociclib + fulvestrant in the INAVO120 clinical trial, patients whose disease is positive for a *PIK3CA* mutation (which is associated with more aggressive disease biology) should be considered for this endocrine-based strategy even with minimal symptoms and a high disease burden.

**Recommendation** **7.** *For patients with HR+/HER2− breast cancer without a PIK3CA mutation and whose disease relapses on or ≤12 months after completion of adjuvant AI, SERD is the preferred ET backbone. [Strong recommendation]*

The ASCO and ESMO guidelines recommend fulvestrant, a SERD, as the ET backbone with recurrence on or within recent exposure to AI therapy [7,9,14]. In Canada, fulvestrant is the current SERD used in practice. However, REAL Alliance acknowledges that newer generation oral SERDs may soon have Health Canada approval and should then be considered as the ET backbone based on recent data from clinical trial (Please see Recommendation 17) [26].

**Recommendation** **8.** *For patients with HR+/HER2− breast cancer without a PIK3CA mutation and whose disease relapses on or ≤12 months after completion of adjuvant AI, and without prior adjuvant CDK4/6i, the standard of care treatment is either ribociclib + fulvestrant or abemaciclib + fulvestrant. In the case of contraindications or intolerance, palbociclib + fulvestrant may be considered. [Strong recommendation]*

With the OS benefit seen in phase 3 trials, ribociclib or abemaciclib in combination with a SERD are recommended as the standard of care for women (irrespective of menopausal status) or men with HR+/HER2− metastatic breast cancer, whose disease relapses on or ≤12 months after completion of adjuvant ET. The decision regarding which treatment to select depends on the totality of evidence associated with each drug as well as patient preferences regarding expected toxicities of available agents.

The MONALEESA-3 trial demonstrated that ribociclib + fulvestrant significantly improved OS compared to fulvestrant alone in postmenopausal women and men with HR+/HER2− advanced breast cancer, including those whose disease relapsed on or ≤12 months from completion of (neo)adjuvant ET. In the overall population, the combination of ribociclib + fulvestrant reduced the risk of death by 28%, extended median OS by 12.2 months, and increased the estimated 5-year survival rate from 31% to 46% compared to fulvestrant alone [52,53] [ESMO-MCBS v2.0 score: 4].

MONARCH-2 showed that abemaciclib + fulvestrant significantly improved OS in women whose disease had progressed on (neo)adjuvant ET, ≤12 months after adjuvant ET, or while receiving ET for advanced breast cancer. In the final survival analysis, combination therapy reduced the risk of death by 22% and extended median OS by 8.6 months compared to fulvestrant alone. The 5-year OS was also longer with abemaciclib + fulvestrant vs. fulvestrant alone (41.2% vs. 29.2%, respectively) [54] [ESMO-MCBS v2.0 score: 3].

Clinical evidence from the PALOMA-3 study supports the recommendation that palbociclib can be considered in patients who have contraindications or intolerance to both ribociclib and abemaciclib, despite the lack of OS benefit [55] [ESMO-MCBS v2.0 score: 4].

**Recommendation** **9.** *For patients with HR+/HER2− breast cancer without a PIK3CA mutation and whose disease relapses on or ≤12 months after completion of adjuvant AI, and with prior adjuvant CDK4/6i, rechallenge with ET + CDK4/6i should be considered, depending on the clinical situation and timing of relapse. [Moderate recommendation]*

As CDK4/6i are now used in the adjuvant setting, it is important to consider both the timing of relapse in relation to ET and the prior use of CDK4/6i when selecting first-line metastatic therapy, though there are limited data for patients whose disease relapses after treatment with adjuvant CDK4/6i.

The phase 3 EMBER-3 study evaluated imlunestrant (an oral SERD) either alone or in combination with abemaciclib compared to ET (fulvestrant or AI) monotherapy in patients whose disease had recurred or progressed during or after treatment with an AI +/–CDK4/6i in the neoadjuvant, adjuvant, or first-line advanced breast cancer setting [56]. Median PFS was 9.4 vs. 5.5 months with imlunestrant + abemaciclib vs. imlunestrant monotherapy (HR 0.57, 95% CI 0.44–0.73, *p* < 0.001), with an updated analysis showing a trend in improved OS with ET + CDK4/6i [57] [ESMO-MCBS v2.0 score: 3].

The phase 2 MAINTAIN and phase 3 postMONARCH studies also evaluated rechallenge with ET and CDK4/6i, mostly in patients progressing in the metastatic setting [58,59]. In MAINTAIN, rechallenge with ET + ribociclib delayed progression by 2.5 months, doubling median PFS from 2.76 to 5.29 months [58], and in postMONARCH, rechallenge with ET + abemaciclib resulted in a modest overall clinical benefit (median PFS 6.0 vs. 5.3 months) [59]. Limitations of these studies must be considered when applying these data to clinical practice. The benefits of rechallenge with CDK4/6i were most notable for patients who received palbociclib in the first-line setting [56,58,59] and these studies were conducted before targeted therapies were more widely used in the first-line setting. Further, nearly all patients had received their previous CDK4/6i in the advanced setting, and most patients had been treated with a CDK4/6i for more than 12 months [58].

This recommendation of CDK4/6 inhibitor rechallenge is based on limited evidence. Although it is not clear if these data can be extrapolated to rechallenging with a CDK4/6i after adjuvant treatment with ribociclib or abemaciclib, REAL Alliance endorses a moderate recommendation to consider rechallenge with ET + CDK4/6i in the case of relapse on or ≤12 months after completion of adjuvant ET + CDK4/6i depending on the clinical scenario. Shared decision-making and consideration of specific clinical circumstances is essential when selecting CDK4/6 inhibitor rechallenge as a suitable option for a patient. In the absence of supporting data, REAL Alliance suggests that the timing of the relapse and rechallenge should be >6 months after completion of adjuvant CDK4/6i.

There may be clinical situations in which rechallenge with another CDK4/6i would not be recommended such as a high burden of disease, rapid disease progression, or a particularly short time interval from initiation of first-line CDK4/6i to disease progression.

**Recommendation** **10.** *For patients with HR+/HER2− breast cancer with a PIK3CA mutation and whose disease relapses on or ≤12 months after completion of adjuvant AI, inavolisib + palbociclib + fulvestrant is the standard of care. [Strong recommendation]*

Approximately 40% of patients with HR+/HER2− breast cancer have oncogenic *PIK3CA* mutations [60], the presence of which is associated with a worse prognosis [61]. Inavolisib is a selective PI3K inhibitor and degrader of the mutated p110α subunit of the enzyme [62].

The phase 3 INAVO120 trial showed that adding inavolisib to fulvestrant and palbociclib significantly improved median PFS (15.0 vs. 7.3 months) and prolonged median OS (34.0 vs. 27.0 months), with a 33% reduction in the risk of death compared to fulvestrant and palbociclib [63,64]. The most common grade 3/4 AEs were neutropenia (82.6%) and thrombocytopenia (13.7%). Inavolisib was associated with higher rates of hyperglycemia, stomatitis or mucosal inflammation, gastrointestinal side effects (such as diarrhea), and ocular side effects (such as dry eye and blurred vision) compared to placebo. Of note, patients enrolled in the trial were required to have a fasting blood glucose <7.0 mmol/L and a glycated hemoglobin level <6.0.%. A limitation of the study is that it included few patients previously treated with adjuvant CDK4/6i [ESMO-MCBS v2.0 score: 3]. Based on these results, Health Canada has approved a triple therapy regimen with inavolisib in combination with fulvestrant + palbociclib for patients with endocrine-resistant, *PIK3CA*-mutated, HR+/HER2− locally advanced or metastatic breast cancer, following recurrence on or after completing adjuvant ET. The term “endocrine resistant” is consistent with the ESMO definition given the timing of relapse relative to ET [9]. However, these patients are still endocrine-eligible even with a certain degree of endocrine resistance.

Hyperglycemia is a known side effect of PI3K inhibitors, even in patients without a history of diabetes, as PI3K mediates the actions of insulin [65]. In the INAVO120 trial, hyperglycemia occurred in 63.4% (any grade) and 6.8% (grade 3/4) of patients treated with inavolisib [63]. Hyperglycemia secondary to PI3K inhibition must be carefully monitored. Baseline and routine assessment of blood glucose levels is recommended for all patients and prophylaxis with metformin can be considered for patients considered to be at high risk of developing hyperglycemia (e.g., (pre)diabetes, age ≥ 45 years, BMI ≥ 30 kg/m^2^, family history of diabetes, history of gestational diabetes) [62,66].

Combinations of inavolisib + fulvestrant with other CDK4/6is (abemaciclib, ribociclib) are currently under investigation. The MORPHEUS-panBC umbrella study (NCT03424005) is evaluating multiple treatment combinations in patients with advanced/metastatic breast cancer [67]. After a median duration of follow-up of 6 months, preliminary results in the 12 patients with *PIK3CA* mutated, HR+/HER2− advanced breast cancer who received inavolisib + fulvestrant in combination with ribociclib (*n* = 6) or abemaciclib (*n* = 6) showed manageable safety profile. However, this study is still ongoing, and more data is required in larger populations before such combinations can safely be employed in the clinic [67].

Hence, the current standard of care is inavolisib in combination with palbociclib + fulvestrant in the appropriate patient, after consideration of patient baseline glycemic control, treatment goals, and benefit-to-risk profile.

### 3.4. First-Line Metastatic Treatment Recommendations for Special Populations

Table 4 summarizes REAL Alliance recommendations for first-line treatment of special populations with metastatic breast cancer.

**Recommendation** **11.** *For patients with HR+/HER2− metastatic breast cancer and limited life expectancy or for those who, with shared decision-making, wish not to have intensive monitoring or toxicities, ET alone is a reasonable option. [Expert opinion]*

In the context of metastatic breast cancer, limited life expectancy may arise either from the underlying diagnosis, recognizing that prognosis can be meaningfully modified with appropriate therapy, or from competing comorbid conditions, such as chronic obstructive pulmonary disease or heart failure. The recommendation for ET alone is based on evidence from the phase 3 SONIA study, which evaluated the optimal sequencing of CDK4/6is and ET in patients with HR+/HER2− metastatic breast cancer [69]. The study compared two treatment strategies: (1) first-line CDK4/6i + AI followed by fulvestrant upon progression (CDK4/6i-first group), and (2) first-line AI monotherapy followed by CDK4/6i + fulvestrant upon progression (CDK4/6i-second group). Most (91%) patients received palbociclib as the CDK4/6i. After a median follow-up of 37.3 months, the primary endpoint of PFS on second-line therapy (PFS2) was equivalent between groups (31.0 vs. 26.8 months, respectively; HR 0.87, 95% CI 0.74–1.03, *p* = 0.10). However, median PFS on first-line therapy (PFS1) was significantly longer in the CDK4/6i-first group (24.7 vs. 16.1 months, respectively; HR 0.59, 95% CI 0.51–0.69, *p* < 0.0001). Patients in both groups reported similar physical, social, emotional, and functional well-being but there was a higher incidence of grade ≥3 AEs in the CDK4/6i-first group compared to the CDK4/6i-second group (83% vs. 64%), possibly due to a three-times longer duration of treatment exposure (24.6 vs. 8.1 months, respectively) [69]. In the overall population, first-line CDK4/6i use did not improve median OS compared to second-line use (45.9 vs. 53.7 months, respectively; HR 0.98, 95% CI 0.80–1.20, *p* = 0.83). However, in premenopausal women, there was a significant benefit with first-line CDK4/6i (HR 0.53, 99% CI 0.27–1.02, *p* = 0.01) [70].

Based on the efficacy and safety observed in SONIA, REAL Alliance opinion is that ET alone is a reasonable option in situations when a patient has limited life expectancy or when, after shared decision-making, the patient wishes to avoid intensive monitoring or toxicities associated with CDK4/6i + ET. Broader application of SONIA is limited, as the trial evaluated palbociclib as the CDK4/6i, while ribociclib or abemaciclib are the standard of care in Canadian practice (Recommendation 6).

In the phase 3 FALCON study, first-line fulvestrant in patients with HR+/HER2− advanced breast cancer showed a trend for improved OS compared to anastrozole in patients with bone-only disease (median OS 65.2 vs. 47.8 months, respectively; HR 0.85, 95% CI 0.60–1.20) [71] [ESMO-MCBS v2.0 score: 2]. With emerging data from the EMBER-3 trial, imlunestrant may be appropriate in certain populations (Recommendations 16 and 17) [56,57].

**Recommendation** **12.** *For eligible older patients (e.g., ≥75 years of age) with HR+/HER2− metastatic breast cancer, the standard of care at standard recommended doses remains the same as that for younger patients. [Strong recommendation]*

Over 40% of metastatic breast cancer diagnoses and recurrences occur in people aged 70 years and older [72], a population often underrepresented in clinical studies [73]. A small number of older patients were enrolled in MONALEESA, MONARCH, and PALOMA trials and the clinical benefit of CDK4/6i + ET was consistent across all age groups, including older patients aged ≥ 75 years [74,75,76]. Furthermore, a meta-analysis of 10 clinical trials concluded that the addition of CDK4/6i to ET significantly reduced mortality in both younger and older patients (i.e., those ≥65 years) [77].

There is often a missed opportunity to individualize dosing in older patients. In MONALEESA-2, and -3, ribociclib + ET discontinuation rates due to AEs were numerically higher in older patients and these patients discontinued their treatment without dose reduction more often than younger patients [74]. The efficacy of therapy, however, can be maintained with CDK4/6i despite dose reductions [78]. For older patients who meet eligibility criteria, starting their therapy at standard doses remains the optimal approach as it maximizes therapeutic efficacy while maintaining an acceptable safety profile [79]. REAL Alliance recommends dose reductions as needed to manage AEs.

The phase 2 AMALEE study demonstrated that ribociclib at a starting dose of 400 mg did not achieve noninferiority to the 600 mg starting dose for overall response rate (ORR); ORR was 48.9% with ribociclib 400 mg vs. 56.1% with ribociclib 600 mg with the lower boundary of the 90% confidence interval less than the prespecified noninferiority margin [80]. However, duration of response (26.5 vs. 28.8 months) and median PFS (26.9 vs. 25.1 months) were comparable among patients receiving the 400 mg vs. 600 mg dose of ribociclib. Time to response was longer with the lower dose of ribociclib (13.1 vs. 9.0 months); however, QTcF prolongation was reduced, and the rates of grade 3/4 neutropenia (41.0% vs. 58.5%) were lower. These data confirm the 600 mg ribociclib starting dose and support the management of dose-dependent AEs with dose reductions.

When carefully selected based on overall health status and functional capacity rather than chronological age alone, older patients can tolerate and benefit from standard dosing regimens like their younger counterparts, a concept that reinforces the importance of individualized treatment decisions to ensure patients receive the most effective therapy.

**Recommendation** **13.** For patients with HR+/HER2− metastatic breast cancer and visceral disease and in the absence of true visceral crisis*, ribociclib + ET or abemaciclib + ET is standard of care (instead of chemotherapy) with close monitoring for progression of disease or lack of response. Palbociclib + ET may be considered if neither ribociclib nor abemaciclib are suitable. [Strong recommendation]

* Defined as severe organ dysfunction, as assessed by signs and symptoms, laboratory studies and rapid progression of disease. For example: rapidly increasing dyspnea at rest in the absence of pleural effusion in the lungs or rapidly increasing bilirubin >1.5× upper limit of normal (ULN) in the absence of an obstruction in the liver [68].

The phase 2 RIGHT Choice study was the first randomized trial comparing CDK4/6i + ET vs. combination chemotherapy in premenopausal women with clinically aggressive HR+/HER2− advanced breast cancer (i.e., defined as symptomatic visceral metastases, rapid disease progression or impending visceral compromise, or markedly symptomatic non-visceral disease) [81]. Two-thirds of patients had symptomatic visceral metastases, and 18.5% experienced rapid disease progression/impending visceral compromise. Visceral crisis was documented in 47.7% of the study population and was defined according to the former 3rd ESO–ESMO International Consensus Guidelines for Advanced Breast Cancer (ABC3) [82]. Ribociclib + ET vs. combination chemotherapy demonstrated significantly superior PFS (21.8 vs. 12.8 months), a similar response rate (66% vs. 62%), and improved tolerability. The benefit was less pronounced in patients experiencing a visceral crisis and in those with recurrent disease. Although the multi-agent chemotherapy comparator arm does not reflect the standard of care in Canada, which is to use single-agent chemotherapy in visceral disease in the absence of visceral crisis, this study nonetheless supports the use of first-line ribociclib + ET vs. combination chemotherapy in patients with aggressive disease, particularly in the absence of visceral crisis [81].

In the phase 2 ABIGAIL study, first-line abemaciclib + ET demonstrated a superior early response compared to single-agent paclitaxel in patients with HR+/HER2− advanced breast cancer at risk of rapid progression due to aggressive disease (defined as the presence of ≥1 of the following: visceral metastases, grade 3 and/or negative PR on primary tumour, lactate dehydrogenase >1.5× ULN, and/or disease progression on or within 36 months after completing adjuvant ET) [83]. The primary endpoint of 12-week ORR was 59% with abemaciclib + ET and 40% with paclitaxel (odds ratio 2.12, 95% CI 1.13–3.96, *p* = 0.019) [83], supporting the benefit of this approach over standard frontline chemotherapy in patients with aggressive disease. The study is ongoing to assess additional efficacy outcomes, including PFS [83]. While the ABIGAIL study enrolled a lower-risk population than the RIGHT Choice trial, the use of single-agent chemotherapy as the comparator is consistent with Canadian clinical practice.

Palbociclib + ET is an option for patients with HR+/HER2− metastatic breast cancer and visceral disease if treatment with ribociclib and abemaciclib is considered unsuitable. The phase 3 PEARL study in postmenopausal, AI-resistant women with metastatic breast cancer demonstrated palbociclib + ET was safer and better preserved quality of life compared to single-agent capecitabine, without a significant PFS benefit [84]. The phase 2 Young-PEARL study in pre/perimenopausal, tamoxifen-resistant women with metastatic breast cancer showed palbociclib + ET prolonged median PFS by 5.7 months vs. capecitabine, although the study excluded individuals with symptomatic serious visceral metastases [85].

These studies support the use of first-line CDK4/6i + ET for patients with HR+/HER2− aggressive and/or visceral metastatic disease in the absence of visceral crisis.

**Recommendation** **14.** *For patients with HR+/HER2− metastatic breast cancer with bone metastases, the use of bone-modifying agents (e.g., bisphosphonates or denosumab) is standard of care to reduce and delay skeletal related adverse events. [Strong recommendation]*

Bone metastases occur in up to 70% of patients with metastatic breast cancer [86,87]. Patients can experience skeletal-related events (SREs), particularly pain and structural damage, that lead to reduced daily functioning and poor quality of life. Delaying the time to first skeletal-related AEs protects patients from serious bone complications, reduces bone pain, and improves quality of life [88]. Bone-modifying agents (e.g., bisphosphonates, denosumab) are recommended for individuals with bone metastases, irrespective of whether patients are symptomatic [86,89].

The dosing interval of bone-modifying agents should follow recommended guidelines [86]. For example, the EMSO guideline recommends administering zoledronate every 12 weeks for patients with stable disease following 3 to 6 months of monthly treatment, or denosumab every 4 weeks. Denosumab has demonstrated superior efficacy compared to zoledronate in delaying both initial and subsequent SREs; however, it has a higher risk of osteonecrosis of the jaw [88]. After 2 years of treatment, de-escalating to a once every 24 weeks dosing schedule may be appropriate. REaCT-Hold BMA showed that this approach preserved physical function without increasing functional interference, with comparable rates of symptomatic skeletal events and HRQoL [90]. Due to the risk of osteonecrosis of the jaw associated with bisphosphonates, dental evaluation and preventive dentistry are recommended prior to initiating therapy. Calcium and vitamin D supplementation is also advised [86,89].

**Recommendation** **15.** *For patients with HR+/HER2− metastatic breast cancer with CNS involvement, a multidisciplinary team should be involved in providing recommendations for optimal local and systemic therapies. Currently, there is insufficient evidence to recommend any given systemic therapy alone (in the absence of local therapy) for the treatment of “active” HR+/HER2− CNS metastases. [Moderate recommendation]*

In contrast to the HER2+ space where systemic therapies for central nervous system (CNS) metastases have demonstrated efficacy with potential to spare local therapy, effective systemic therapies for HR+/HER2− CNS metastases are still lacking [91]. This is particularly relevant given a substantial risk of CNS metastases among patients with HR+/HER2− breast cancer [92], which is magnified among patients with endocrine-resistant disease who undergo MRI-based screening for CNS metastases [93]. Therefore, multidisciplinary care is critical, with most patients still requiring local treatment for HR+/HER2− CNS metastases.

Currently, with CDK4/6is, the literature for CNS metastases has mainly focused on abemaciclib, which has been shown to penetrate the blood brain barrier [94,95]. In a phase 2, single-arm trial (NCT02308020), abemaciclib as monotherapy at a higher dose of 200 mg twice daily or with ET demonstrated a clinical benefit in a subset of patients (*n* = 58) with HR+/HER2− metastatic breast cancer and CNS metastases. Eligible patients had ≥1 measurable brain lesion (≥10 mm by RANO-BM criteria) that was “active”, defined as, either new or not previously irradiated, or progressive after prior irradiation. Intracranial lesion size decreased in 38% of patients and the intracranial clinical benefit rate was 24.1% [96]. However, the trial failed to meet its primary endpoint of intracranial ORR.

In the phase 3b CompLEEment-1 trial, a subgroup analysis of 51 patients with HR+/HER2− advanced breast cancer and previously treated or stable CNS metastases showed that ribociclib + letrozole achieved time to progression, ORR, and clinical benefit rate that were consistent with those observed in the overall study population [97]. However, intracranial efficacy was not assessed in this study because patients with active brain metastases were not included. In the phase 3 EMBER-3 study, CNS progression in patients with previously treated and stable CNS metastases was found to be numerically less frequent with imlunestrant compared to ET monotherapy, although the number of events was small [56]. Currently, the CNS activity of imlunestrant + abemaciclib is unknown given that patients with active CNS metastases were not included in EMBER-3.

PI3K inhibitors have also shown potential CNS activity based on case reports [98], but clinical trial data is currently lacking.

In DESTINY-BREAST04, T-DXd showed promise in patients with HER2-low breast cancer and stable, previously treated brain metastases [99]. The phase 2, single-arm TUXEDO-4 study (NCT06048718) is underway to evaluate T-DXd in patients with HER2-low breast cancer and active brain metastases [100]. Other ADCs, including sacituzumab govitecan and patritumab deruxtecan (HER3-DXd), have demonstrated CNS activity in HR+/HER2− metastatic breast cancer with brain metastases [101,102], with additional trials evaluating efficacy of datopotamab-deruxtecan (dato-DXd) still ongoing (NCT06176261, NCT06533826). Standard chemotherapies, particularly capecitabine and platinum-based regimens, have also shown intracranial activity in breast cancer CNS metastases, and are listed as CNS-active agents in National Comprehensive Cancer Network (NCCN) guidelines [103,104,105].

### 3.5. Second-Line Metastatic Treatment for Patients with Endocrine-Eligible Breast Cancer (Progression on ET in the Metastatic Setting)

With the growing treatment landscape and advances in genomic testing, the optimal sequencing of ET and targeted therapies in the second-line metastatic setting continues to rapidly evolve. Table 5 summarizes REAL Alliance recommendations for second-line treatment of patients with endocrine-eligible metastatic breast cancer.

**Recommendation** **16.** *For patients with HR+/HER2− metastatic breast cancer with no targetable mutations whose disease progresses on first-line therapy of ET + CDK4/6i, switching ET + another CDK4/6i could be considered. Everolimus + ET is another consideration. [Moderate recommendation]*

The phase 2 MAINTAIN and phase 3 post-MONARCH showed the benefit of CDK4/6i + ET rechallenge after progression on CDK4/6i + ET [58,59] (See Recommendation 9). In the phase 2 PACE study, palbociclib + fulvestrant was not superior to fulvestrant alone after progression on CDK4/6i + AI (median PFS 4.6 vs. 4.8 months, respectively; HR 1.11, 90% CI 0.79–1.55, *p* = 0.62) [106].

Orals SERDs are a promising treatment option for patients whose disease progresses on first-line ET + CDK4/6i in the metastatic setting [56,107,108]. In the phase 3 EMERALD study, elacestrant significantly prolonged median PFS vs. fulvestrant in the overall population (including patients with and without *ESR1* mutations) with median PFS of 2.8 vs. 1.9 months, respectively (*p* = 0.0019). The magnitude of the benefit, however, was less than observed in patients with a detectable *ESR1* mutation (median PFS 3.8 vs. 1.9 months, respectively; *p* = 0.005) [107] [ESMO-MCBS v2.0 score: 3].

Adding a CDK4/6i to an oral SERD may further improve PFS. As mentioned in Recommendation 9, the phase 3 EMBER-3 study assessed 3 treatment arms (imlunestrant, imlunestrant + abemaciclib, and standard of care ET, which included exemestane or fulvestrant) in patients whose disease recurred or progressed during or after treatment with an AI ± CDK4/6i [56]. The study included patients with or without an *ESR1* mutation. In the overall population, after a median follow-up of 27 months, the median PFS doubled with the addition of abemaciclib to imlunestrant, from 5.5 months with monotherapy to 10.9 months with the combination (HR 0.59, 95% CI 0.47–0.74, *p* < 0.0001). Consistent with this PFS benefit, the median OS was not reached with imlunestrant + abemaciclib vs. 34 months with imlunestrant monotherapy (HR 0.82, 95% CI 0.59–1.16, *p* = 0.2622) [57]. The combination therapy was effective in patients with *ESR1* mutation, as well as in patients without *ESR1* mutation. Recommendation 17 outlines treatment options specifically for patients with *ESR1* mutation.

Several studies have shown the benefit of everolimus + ET in the second-line metastatic setting [108,109,110]. In the phase 3 BOLERO-2 study, everolimus + exemestane resulted in more than a doubling of median PFS compared to exemestane alone (median PFS 7.8 vs 3.2 months; HR 0.45, 95% CI 0.38–0.54, *p* < 0.0001) in patients whose disease had progressed on a NSAI [109]. However, no OS benefit was seen in the study (31.0 vs. 26.6 months; HR 0.89, 95% CI 0.73–1.10, *p* = 0.14) and the toxicity profile can be limiting for some patients (e.g., risk of mucositis, pneumonitis) [ESMO-MCBS v2.0 score: 1].

While the ESMO guidelines recommend everolimus + exemestane after disease progression after first-line ET + CDK4/6i in patients with no risk of organ failure [9], the supporting data predate the CDK4/6i era. In the first prospective study of everolimus + fulvestrant in HR+/HER2− metastatic breast cancer in patients whose disease progressed on prior CDK4/6i, the median PFS was 6.8 months, and median OS was 38.2 months [110].

The benefit of an oral SERD + everolimus in the post-CDK4/6i setting was demonstrated in the phase 3 evERA Breast Cancer trial [108]. Giredestrant + everolimus significantly prolonged PFS vs. standard of care ET + everolimus from 5.49 to 8.77 months in the overall population, reflecting a 44% reduction in the risk of progression or death (HR 0.56, 95% CI 0.44–0.71, *p* < 0.0001); subgroup analysis by *ESR1* and *PIK3CA* mutational status are pending.

**Recommendation** **17.** *For patients with HR+/HER2− metastatic breast cancer with ESR1 mutations (without PIK3CA alterations) whose disease progresses on first-line therapy of AI + CDK4/6i, a SERD is standard of care ET, either as monotherapy or in combination with a targeted agent. [Strong recommendation]*

Among the molecular mechanisms driving ET resistance, *ESR1* mutations are detected in 20% to 40% of patients in the metastatic setting, particularly after AI treatment [111]. Several trials have shown that next-generation oral SERDs or a proteolysis-targeting chimera (PROTAC) agent are effective in overcoming resistance to *ESR1* mutations in patients with HR+/HER2− metastatic breast cancer whose disease had advanced on at least one ET, including ET monotherapy or in combination with CDK4/6i (Table 6) [56,107,108,112,113,114]. Oral SERDs consistently showed better PFS outcomes than standard ET (fulvestrant or AI) and quality of life was improved with oral agents compared to injectable options like fulvestrant [56].

While oral SERDs (elacestrant, imlunestrant, camizestrant, and giredestrant) and vepdegestrant (a PROTAC agent) were modestly more effective than fulvestrant in *ESR1*-mutated breast cancer, their administration orally rather than by intramuscular injection is often preferred by patients. Currently, oral SERDs do not have Health Canada approval.

To optimize outcomes among patients, there may be a benefit of switching from AI to an oral SERD upon detection of an *ESR1* mutation by ctDNA testing prior to disease progression. Results from the ongoing phase 3 SERENA-6 study reported that switching from AI to camizestrant while maintaining the same first-line CDK4/6i (palbociclib, ribociclib or abemaciclib) provided a clinically meaningful PFS benefit with a 66% risk reduction in disease progression (HR 0.44, 95% CI 0.31–0.60, *p* < 0.00001) [26]. Median PFS was 16 months with camizestrant + CDK4/6i vs. 9.2 months with AI + CDK4/6i. Episodes of photopsia (grade 1: 20%; grade 3: 1%) were transient, not associated with changes in optic structure or visual acuity, and for most patients photopsia had no/minimal impact on daily activities [26]. Quality of life data showed a delay to deterioration among patients who switched to camizestrant [115]. However, because only ~14% of patients in the control arm received camizestrant or another oral SERD after progression, the extent to which early implementation at the time of molecular progression affects longer-term outcomes remains to be determined.

**Recommendation** **18.** *For patients with HR+/HER2− metastatic breast cancer whose disease progresses on first-line therapy and whose tumour has an PI3K/AKT/PTEN pathway alteration (with no prior PI3K/AKT/PTEN-directed therapy), standard of care in the second-line setting is capivasertib + fulvestrant. [Strong recommendation] Alpelisib + fulvestrant could be considered for patients with a confirmed PIK3CA mutation. [Moderate recommendation]*

Capivasertib is an oral inhibitor of the PI3K/AKT/PTEN signaling pathway, inhibiting the kinase activity of all three isoforms of serine/threonine kinase AKT (AKT1, AKT2 and AKT3) [116]. The phase 3 CAPItello-291 study demonstrated capivasertib in combination with fulvestrant resulted in significantly longer PFS than ET alone in patients whose disease progressed on or after previous AI therapy [117]. Over two-thirds (69.1%) of the population had prior exposure to CDK4/6i therapy in the advanced setting and 40.8% had tumours with PI3K/AKT/PTEN pathway alterations. At a median follow-up of about 13 months, patients with tumours harboring PI3K/AKT/PTEN pathway-altered mutations experienced the most clinical benefit from capivasertib + fulvestrant, with a median PFS more than double that of standard of care (7.3 vs. 3.1 months; HR 0.50, 95% CI 0.38–0.65, *p* < 0.001). Quality of life and global health status were maintained for twice as long with capivasertib + fulvestrant than with placebo + fulvestrant (24.9 months vs. 12 months, respectively) [117], except for diarrhea, which worsened for patients on capivasertib [118] [ESMO-MCBS v2.0 score: 2].

Similar results have been observed in the phase 3 CCTG/BCT MA.40/FINER study evaluating fulvestrant + ipatasertib for treatment of HR+/HER2− breast cancer after progression on first-line CDK4/6i + AI [119].

Alpelisib is an oral α-selective PI3K inhibitor and degrader [120]. In the phase 3 SOLAR-1 study in postmenopausal women or men, alpelisib + fulvestrant prolonged median PFS vs. fulvestrant (11.0 vs. 5.7 months, HR 0.65, 95% CI, 0.50–0.85, *p* = 0.00065) with no statistically significant OS benefit [121,122] [ESMO-MCBS v2.0 score: 1]. Notably, only 5.9% of patients had received prior CDK4/6i therapy in SOLAR-1. The phase 2, single-arm BYLieve trial showed that alpelisib + fulvestrant was effective in 127 patients whose immediate prior treatment was CDK4/6i + AI [123]. After a median follow-up of 21.8 months in 119 patients with *PIK3CA* mutations, median PFS was 8.0 months, and median OS was 27.3 months. Results of a confirmatory phase 3 trial (INAVO121; inavolisib + fulvestrant vs. alpelisib + fulvestrant) post-CDK4/6 inhibitor are pending (NCT05646862).

Diarrhea, rash, nausea, and hyperglycemia are among the most common AEs associated with capivasertib and alpelisib and toxicity associated with *PIK3CA* pathway inhibitors can often lead to dose interruptions or treatment discontinuation [117,122,124]. Hyperglycemic events, which tend to be more frequent and more severe with alpelisib, typically occur within the first 3 weeks of treatment [9]. When selecting patients for treatment, comorbidities (e.g., diabetes, pre-diabetes) must be considered given the potential risk of toxicities, such as hyperglycemia, diarrhea, or rash [7,9].

### 3.6. Third-Line Metastatic Treatment for Patients with Endocrine-Eligible Breast Cancer (Progression on ET in the Metastatic Setting)

Table 7 summarizes REAL Alliance recommendations for third-line treatment in endocrine-eligible patients with metastatic breast cancer.

**Recommendation** **19.** *For patients who have had two prior lines of ET (without CDK4/6i) in the metastatic setting and whose disease progresses, a trial of CDK4/6i + ET using a different ET agent should be considered. [Moderate recommendation]*

In endocrine-eligible patients in the third-line metastatic setting, adding a CDK4/6i + sequential use of endocrine-based therapies (i.e., an ET agent not used in a prior line) could delay chemotherapy and may provide some clinical benefit [9]. As most patients receive CDK4/6i as their first-line therapy, scenarios in which a patient will have had two prior lines of ET without a CDK4/6i in the metastatic setting are infrequent in clinical practice.

Limited clinical data are available in patients whose disease progressed after second-line or third-line ET. MONARCH-2 and MONALEESA-3, however, included patients whose disease progressed on up to one line of ET in the metastatic setting [125,126]. Prior treatment with fulvestrant was an exclusion criterion in these studies, underscoring the importance of switching the ET backbone when adding CDK4/6i therapy after progression on ET in the metastatic setting.

### 3.7. Metastatic Treatment of Patients with Endocrine-Ineligible Breast Cancer

Table 8 summarizes REAL Alliance recommendations for treatment of patients with endocrine-ineligible metastatic breast cancer.

**Recommendation** **20.** *For patients with HR+/HER2− metastatic breast cancer that is ET ineligible and has progressed after prior ET + CDK4/6i in any setting, chemotherapy, including ADC, is the standard of care. [Strong recommendation]*

Non-ET-based chemotherapy for patients who are endocrine-ineligible include conventional chemotherapy or ADC. Single-agent chemotherapy is generally recommended though a doublet regimen may be considered in cases of aggressive disease or visceral crisis, where a rapid response is required [9,14,127]. The choice of chemotherapy should consider the patient’s prior exposure to chemotherapy, features of their disease (e.g., location and burden of disease, pace of progression), safety profile of the proposed regimen, patients’ comorbidities, and patient preferences.

Capecitabine or another single-agent chemotherapy (e.g., taxane, eribulin, gemcitabine) may be used based on shared decision-making, patient preference, and clinical considerations, recognizing the manageable toxicity profiles and oral convenience that some patients value. Under the “treatment-by-physician’s choice” category used in pivotal ADC trials, these agents remain appropriate options when ADCs are not suitable. Capecitabine is often preferred given that it is an oral agent with a reasonable toxicity profile. Before starting capecitabine treatment, patients should undergo germline testing to detect variants associated with dihydropyrimidine dehydrogenase (DPD) deficiency [128]. Patients with little or no DPD enzyme activity, due to certain mutations in the *DPYD* gene, are at significantly increased risk of severe, potentially fatal reactions to fluorouracil. Those with complete or near-complete DPD deficiency should not be treated with capecitabine, as no safe dose has been established for this population.

T-DXd has become a key standard of care in HER2-low metastatic breast cancer across multiple lines of therapy. In patients who had received prior chemotherapy and ≥1 prior ET, DESTINY-Breast04 demonstrated both PFS and OS benefit supporting its routine use. More recently, DESTINY-Breast06 showed superiority of T-DXd as first chemotherapy after ≥1 prior lines of ET compared to standard of care chemotherapy and has received Health Canada approval. As OS data for DESTINY-Breat06 continues to mature, these results further support the role of T-DXd earlier in the treatment pathway. Additional context regarding the positioning of T-DXd in the first-line setting (Recommendation 21), second-line setting (Recommendation 23), and beyond, including the use of sacituzumab govitecan in later-line settings is provided in subsequent recommendations [129].

**Recommendation** **21.** *(a) For patients with HR+/HER2-low or ultralow metastatic breast cancer that has progressed on prior ET + CDK4/6i, T-DXd is an option as the next line of systemic therapy if no prior chemotherapy has been given, guided by shared decision-making. [Strong recommendation] (b) For patients with HER2-low disease who have not previously received T-DXd, but who have received at least one line of chemotherapy, T-DXd is standard of care. [Strong recommendation]*

A spectrum of HER2 expression exists among tumours considered negative for HER2 expression. About 65% of HR+/HER2− breast cancer tumours express low levels of HER2 (HER2-low) [130]. HER2-low status is defined as membrane staining with an IHC score of 1+ or 2+ with a lack of amplification by in situ hybridization [22,131]. In the scenario of incomplete or faint membrane staining (IHC > 0 and <1+) in ≤10% of invasive cancer cells, tumours are defined as HER2-ultralow [22].

The phase 3 DESTINY-Breast06 trial demonstrated T-DXd significantly prolonged PFS compared to physician’s choice of chemotherapy (13.2 vs. 8.1 months; HR 0.62, 95% CI 0.51–0.74, *p* < 0.001) in patients with HR+/HER2-low/ultralow metastatic breast cancer who had received at least 1 prior line of ET in the metastatic setting [129]. All patients were chemotherapy naïve in the metastatic setting and most (89%) had received prior ET + CDK4/6i. In both the intention-to-treat (HR+/HER2-low/ultralow) and HER2-ultralow populations, T-DXd prolonged PFS vs. traditional chemotherapy. Exploratory biomarker analysis by ctDNA showed that T-DXd improved outcomes vs. chemotherapy regardless of genomic status for patients with *AKT*, *PIK3CA*, *PTEN*, *ESR1*, or *BRCA1/2* mutations [132]. T-DXd significantly improved patient-reported outcomes, leading to notable improvements in symptom control, particularly with respect to pain and fatigue, and a significant increase in overall HRQoL compared to chemotherapy [ESMO-MCBS v2.0 score: 3]. Furthermore, patients receiving T-DXd reported higher levels of treatment satisfaction. These findings underscore the potential of T-DXd not only to provide superior clinical efficacy but also to improve critical aspects of patient well-being, highlighting its value as a therapeutic option for this patient population.

The pivotal phase 3 DESTINY-Breast04 study was the first to show that targeting T-DXd in patients with HER2-low advanced breast cancer led to statistically significant and clinically meaningful benefits in PFS and OS compared to conventional, untargeted chemotherapy [99]. Eligible patients had received prior chemotherapy for metastatic disease or had disease recurrence no more than 6 months after completing adjuvant chemotherapy. The HR+ cohort of patients must have progressed on at least one line of ET (with or without a CDK4/6i) and were deemed ineligible for further ET. Patients had received a median of three lines of treatment for metastatic disease and most (70%) had received a CDK4/6i. T-DXd significantly prolonged median PFS from 5.4 months to 10.1 months compared to physician’s choice chemotherapy (HR 0.51, 95% CI 0.40–0.64, *p* < 0.001) and significantly reduced the risk of death by 36% (median OS: 23.9 vs. 17.5 months; HR 0.64, 95% CI, 0.48–0.86, *p* = 0.003) [ESMO-MCBS v2.0 score: 4]. Patients receiving T-DXd experienced some form of ILD or pneumonitis, with the majority being grade 1 or 2 [99]. Three patients (0.8%) experienced grade 5 ILD. Thus, proactive monitoring of symptoms, prompt management, and risk mitigation are essential to minimize the risk of serious outcomes in patients treated with T-DXd [133]. In addition, T-DXd as an IV therapy and patient preference for IV vs. oral therapy also needs to be considered through shared decision-making.

Pathologists with expertise in breast cancer must carefully review biopsy tissue to identify the subset of patients with HER2-low status. Increased awareness and adequate training of pathologists and oncologists, along with applying standardized scoring criteria and adopting higher-sensitivity assays will be important to refine the existing pathology infrastructure to provide additional clarity on distinguishing HER2-low/ultralow from truly HER2– disease based on the most recent ASCO-College of American Pathologists definitions [22,131]. If HER2-low/ultralow is detected in any biopsy sample (primary or metastatic), patients are eligible to receive T-DXd. If a patient is classified as having HER2– 0 breast cancer, a re-review of the sample by a pathologist is recommended (particularly if HER2– 0 status was assigned historically); if HER2– 0 status is confirmed, physicians should consider obtaining another biopsy.

**Recommendation** **22.** *For patients with HR+/HER2− metastatic breast cancer with germline BRCA1/2 mutations who are no longer benefiting from ET, an oral PARP inhibitor should be considered post-ET instead of chemotherapy. [Strong recommendation]*

An estimated 5% to 10% of people with HR+/HER2− metastatic breast cancer harbor germline breast cancer susceptibility gene (*BRCA1* and *BRCA2*) mutations [134]. As the *BRCA1/2* genes encode proteins involved in homologous-related DNA repair, breast cancers associated with mutations in *BRCA1/2* genes are generally responsive to treatments that inhibit the poly-ADP ribose polymerase (PARP)-mediated DNA repair pathway. Olaparib and talazoparib are PARP inhibitors (PARPi) recommended by international guidelines for the treatment of metastatic breast cancer in patients with germline *BRCA1/2* mutations [9].

Based on clinical evidence from the phase 3 OlympiAD and EMBRACA studies, REAL Alliance recommends evaluating patients with HR+/HER2− metastatic breast cancer for *BRCA1/2* mutation status to identify candidates for PARPi.

The phase 3 OlympiAD study demonstrated the clinical benefit of the PARPi olaparib compared to standard therapy [135,136]. In 302 patients with germline *BRCA* mutation and HER2– metastatic breast cancer (~50% HR+ and 50% triple negative) who had received ≤2 prior chemotherapy regimens in the metastatic setting, median PFS was 7.0 months with olaparib vs. 4.2 month with physician’s choice of single-agent, non-platinum chemotherapy (capecitabine, vinorelbine, or eribulin) (HR 0.58, 95% CI 0.43–0.80, *p* < 0.001) [135]. Despite no significant difference in OS between olaparib and standard therapy (median OS 19.3 vs. 17.1 months, respectively), the 3-year OS was 40.8% vs. 12.8% with first-line use in patients who had not received prior chemotherapy in the metastatic setting [136] [ESMO-MCBS v2.0 score: 4].

Similarly, the phase 3 EMBRACA study showed prolonged PFS with talazoparib vs. chemotherapy (median PFS 8.6 vs. 5.6 months, HR 0.54, 95% CI 0.41–0.71, *p* < 0.0001) but without a significant improvement in OS (median OS 19.3 vs. 19.5 months) [137,138] [ESMO-MCBS v2.0 score: 3].

Randomized trials have not directly compared PARP inhibitors to taxanes, anthracyclines, or platinum salts and the comparative efficacy of these treatments is unknown.

Several other genes function in the homologous-related DNA repair pathway, and the use of PARPi in populations beyond patients with germline *BRCA1/2* mutations has shown promise. The phase 2 TBCRC 048 (Olaparib Expanded) study demonstrated that olaparib is effective in people with metastatic breast cancer and somatic *BRCA1/2* mutations or germline/somatic *PALB2* mutations [139]. The study enrolled 54 patients and 76% had HR+/HER2− breast cancer. Prolonged median PFS was observed among those with germline *PALB2* (13.3 months) and somatic *BRCA1/2* mutations (6.3 months).

While olaparib and talazoparib are Health Canada approved for the treatment of HER2– metastatic breast cancer in patients with *BRCA1/2* mutations [140,141], the drug manufacturers have not filed reimbursement submissions to the Canadian Drug Agency [142], and olaparib and talazoparib are not publicly reimbursed at the time of publication. Olaparib can be accessed through private insurance, direct payment, or patient support programs offered by the manufacturer. There is currently no public or private access to talazoparib in Canada.

**Recommendation** **23.** *For patients with HR+/HER2− metastatic breast cancer that has progressed and who have received ≥2 chemotherapy regimens and no prior ADC, sacituzumab govitecan is the standard of care. [Strong recommendation]*

TROPICS-2 was a phase 3, open-label randomized study that evaluated the trophoblast cell-surface antigen 2 (trop-2)-directed ADC, sacituzumab govitecan, in endocrine-resistant, heavily pretreated patients with HR+/HER2− advanced breast cancer [143,144]. Eligible patients had 2–4 prior lines of chemotherapy and had received at least one prior line of ET including prior CDK4/6i therapy and had exposure to a taxane in any setting. No patients had received an ADC prior to enrolment. All patients included in the study had HER2– disease defined according to ASCO–College of American Pathologists criteria [131], which included those with HER2-low status. At a median follow-up duration of 10.2 months, median PFS was 5.5 months with sacituzumab govitecan and 4.0 months with chemotherapy (HR 0.66, 95% CI 0.53–0.83, *p* = 0.0003) [143]. At the protocol-specified final OS analysis (median follow-up of 12.5 months), sacituzumab govitecan demonstrated a clinical meaningful 3.2-month improvement in OS (median OS 14.4 months with sacituzumab govitecan vs. 11.2 months with chemotherapy) [144]. The most common grade ≥3 AEs with sacituzumab govitecan were neutropenia, diarrhea, leukopenia, anemia, fatigue, and febrile neutropenia [143] [ESMO-MCBS v2.0 score: 4]. Given a high risk of neutropenia with potential for life-threatening febrile neutropenia across breast cancer indications, granulocyte colony-stimulating factor use should be considered either prophylactically or at the time of development of neutropenia.

Datopotamab deruxtecan (dato-DXd) and sacituzumab tirumotecan (sac-TMT) are emerging therapeutic options based on phase 3 evidence [145,146], though the TROPION-Breast01 study improved the median PFS by only 2 months and the OS data is immature [ESMO-MCBS v2.0 score: 3], and the OptiTROP-Breast02 trial requires validation in a broader geographic population.

There is no clear consensus on the optimal sequencing of ADCs. For patients whose disease progresses after prior ADC, the ESMO guidelines state that “sacituzumab govitecan could be considered for patients with HR+/HER2-low/ultralow metastatic breast cancer after prior T-DXd” and, in the event of disease progression, T-DXd may be used after sacituzumab govitecan, if not used in a prior line of therapy [9,14]. There are only limited data, however, on sequencing of one ADC after another. One multicentre, retrospective, French study in heavily pretreated patients found modest outcomes and no significant difference in median PFS2 for patients treated with T-DXd after sacituzumab govitecan (3.1 months) or sacituzumab govitecan after T-DXd (2.2 months) [147]. Median PFS2 was similar regardless of when the next ADC was used (i.e., immediately after the prior ADC or with an intermediate line of chemotherapy between ADCs). A subset of patients benefited from a second ADC for more than 6 months. A multicentre, retrospective cohort study conducted in the US evaluated 84 patients with HER2– metastatic breast cancer treated sequentially with T-DXd and sacituzumab govitecan [148]. The median time to treatment failure was longer with the first ADC than with the second ADC, although there were some patients who experienced notably longer duration of response on their second ADC.

REAL Alliance expert opinion for patients whose disease progresses on an ADC is to enrol them in clinical trials or use another non-ADC chemotherapeutic agent before a trial of a second ADC agent. The uncertainty surrounding whether progression or resistance develops due to the target, the payload, or both highlights the need for strategic treatment options and further research regarding mechanisms of resistance to these agents.

While some patients may maintain their performance status and derive benefit from a second ADC, shared decision-making and consideration of patient factors and preferences is imperative when selecting subsequent treatment after progression on an ADC.

## 4. Conclusions

REAL Alliance consensus recommendations provide a comprehensive, evidence-based framework for the systemic treatment of HR+/HER2− metastatic breast cancer. Recommendations categorize patients according to endocrine sensitivity, timing of relapse, and genomic alterations to support personalized treatment decisions.

Despite recent advances in therapeutic options, challenges remain in treatment sequencing, managing endocrine resistance, and addressing special populations such as those with CNS involvement. Continued clinical research and real-world data will be crucial to refine these recommendations and improve outcomes for patients.

## Figures and Tables

**Table 1:** REAL Alliance recommendations for patients with HR+/HER2− metastatic breast cancer, and comparison with those from ESMO and ASCO.

Recommendations for HR+/HER2− Metastatic Breast Cancer Therapy		REAL	ESMO [9,14]	ASCO [17,18,19]
**1**	If feasible, biopsy of a recurrent lesion(s) should be considered at the time of diagnosis to evaluate biomarkers (ER/PR/HER2 status) and confirm a breast cancer diagnosis if the clinical course is not as expected.	Strong recommendation ●●●	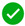	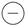
**2**	Testing for somatic pathway alterations (e.g., *PIK3CA*, *PTEN*, *AKT*, *ESR1*) and pathogenic germline variants, (e.g., *BRCA* mutations) is standard of care when it may inform treatment decisions. Testing should be performed with appropriate methods and in a timely manner to guide treatment decisions.	Strong recommendation ●●●	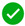	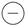
**3**	In the management of HR+/HER2− metastatic breast cancer, it is essential to balance disease control with quality of life, allowing patients to maintain daily activities and minimize treatment-related discomfort.	Strong recommendation ●●●	NC	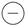
**4**	The treatment and management of breast cancer in men is the same as in pre/perimenopausal women (i.e., ET + CDK4/6i +/– LHRH agonist in the first-line metastatic setting). Given the rarity of breast cancer in men, participation in clinical trials should be encouraged whenever possible.	Strong recommendation ●●●	NC	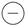

●●●, Strong recommendation; 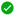, Alignment; 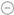, Some variation; NC, Not covered by ESMO and/or ASCO.

**Table 2:** REAL Alliance recommendations for patients with endocrine-sensitive metastatic breast, and comparison with those from ESMO and ASCO.

Recommendations for Patients with Endocrine-Sensitive Metastatic Breast Cancer ^1^		REAL	ESMO [9,14]	ASCO [7]
**5**	**For patients with HR+/HER2− metastatic breast cancer who have not received prior or recent ET**, the standard of care ET backbone in the first-line metastatic setting is an AI.	Strong recommendation ●●●	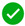	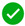
**6**	**For patients with de novo metastatic HR+/HER2− breast cancer, or those whose disease relapses >12 months after completing adjuvant AI, and regardless of adjuvant treatment with CDK4/6i**, the standard of care first-line treatment is CDK4/6i + AI. The preferred CDK4/6i is either ribociclib or abemaciclib; however, in the case of contraindications or intolerance, palbociclib may be considered.	Strong recommendation ●●●	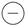	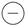

●●●, Strong recommendation; 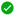, Alignment; 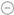, Some variation; NC, Not covered by ESMO and/or ASCO. ^1^ Plus OFS for premenopausal women or LHRH agonist for men.

**Table 3:** REAL Alliance recommendations for patients with endocrine-eligible metastatic breast cancer whose disease relapsed on or ≤12 months after completion of adjuvant AI (first-line metastatic setting), and comparison with those from ESMO and ASCO.

Recommendations for First-Line Treatment of Patients with Endocrine-Eligible Metastatic Breast Cancer ^1^		REAL	ESMO [9,14]	ASCO [7]
**7**	**For patients with HR+/HER2− breast cancer without a *PIK3CA* mutation whose disease relapses on or ≤12 months after completion of adjuvant AI**, SERD is the standard of care ET backbone.	Strong recommendation ●●●	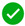	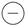 (tumour genomic testing informs second-line treatment)
**8**	**For patients with HR+/HER2− breast cancer without a *PIK3CA* mutation whose disease relapses on or ≤12 months after completion of adjuvant AI, and without prior adjuvant CDK4/6i**, the standard of care treatment is either ribociclib + fulvestrant or abemaciclib + fulvestrant. In the case of contraindications or intolerance, palbociclib + fulvestrant may be considered.	Strong recommendation ●●●	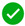 (no CDK4/6i recommended over another)	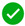 (no CDK4/6i recommended over another)
**9**	**For patients with HR+/HER2− breast cancer without a *PIK3CA* mutation and whose disease relapses on or ≤12 months after completion of adjuvant AI, and with prior adjuvant CDK4/6i**, rechallenge with ET + CDK4/6i should be considered, depending on the clinical situation and timing of relapse.	Moderate recommendation ●●	NC	NC
**10**	**For patients with HR+/HER2− breast cancer with a *PIK3CA* mutation and whose disease relapses on or ≤12 months after completion of adjuvant AI**, inavolisib + palbociclib + fulvestrant is the standard of care.	Strong recommendation ●●●	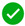	NC

●●●, Strong recommendation; ●●, Moderate recommendation; 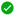, Alignment; 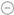, Some variation; NC, Not covered by ESMO and/or ASCO. ^1^ Plus OFS for premenopausal women or LHRH agonist for men.

**Table 4:** REAL Alliance recommendations for the treatment of special populations in the first-line metastatic setting, and comparison with those from ESMO and ASCO.

Recommendations for Special Populations in the First-Line Metastatic Setting ^1^		REAL	ESMO [9,14]	ASCO [7,18]
**11**	**For patients with HR+/HER2− metastatic breast cancer and limited life expectancy or for those who, with shared decision-making, wish not to have intensive monitoring or toxicities**, ET alone is a reasonable option.	Expert opinion ○	NC	NC
**12**	**For eligible older patients (e.g., ≥75 years of age) with HR+/HER2− metastatic breast cancer**, the standard of care at standard recommended doses remains the same as that for younger patients.	Strong recommendation ●●●	NC	NC
**13**	**For patients with HR+/HER2− metastatic breast cancer and visceral disease and in the absence of true visceral crisis ^2^**, ribociclib + ET or abemaciclib + ET is standard of care (instead of chemotherapy) with close monitoring for progression of disease or lack of response. Palbociclib + ET may be considered if neither ribociclib nor abemaciclib are suitable.	Strong recommendation ●●●	NC	NC
**14**	**For patients with HR+/HER2− metastatic breast cancer with bone metastases**, the use of bone-modifying agents (e.g., bisphosphonates or denosumab) is standard of care to reduce and delay skeletal related adverse events.	Strong recommendation ●●●	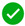	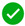
**15**	**For patients with HR+/HER2− metastatic breast cancer with CNS involvement**, a multidisciplinary team should be involved in providing recommendations for optimal local and systemic therapies. Currently, there is insufficient evidence to recommend any given systemic therapy alone (in the absence of local therapy) for the treatment of “active” HR+/HER2− CNS metastases.	Moderate recommendation ●●	NC	NC

●●●, Strong recommendation; ●●, Moderate recommendation; ○, Expert opinion. 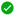, Alignment; NC, Not covered by ESMO and/or ASCO. ^1^ If ET is AI, plus OFS for premenopausal women or LHRH for men; ^2^ The ESO–ESMO International Consensus Guidelines for Advanced Breast Cancer (ABC 6/7) define visceral crisis as “severe organ dysfunction, as assessed by signs and symptoms, laboratory studies and rapid progression of disease” with examples of visceral crisis in the lungs as rapidly increasing dyspnea at rest in the absence of pleural effusion, and in the liver as rapidly increasing bilirubin >1.5× upper limit of normal (ULN) in the absence of an obstruction [68].

**Table 5:** REAL Alliance recommendations for second-line treatment of patients with endocrine-eligible metastatic breast cancer whose disease progresses on ET in the metastatic setting, and comparison with those from ESMO and ASCO.

Recommendations for Second-Line Treatment of Patients with Endocrine-Eligible Metastatic Breast Cancer ^1^		REAL	ESMO [9,14]	ASCO [6,7]
**16**	**For patients with HR+/HER2− metastatic breast cancer with no targetable mutations whose disease progresses on first-line therapy of ET + CDK4/6i**, switching ET + another CDK4/6i could be considered. Everolimus + ET is another consideration.	Moderate recommendation ●●	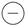 Includes fulvestrant monotherapy	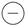 Includes fulvestrant monotherapy
**17**	**For patients with HR+/HER2− metastatic breast cancer with *ESR1* mutations (without *PIK3CA* alterations) whose disease progresses on first-line therapy of AI + CDK4/6i**, a SERD is standard of care ET, either as monotherapy or in combination with a targeted agent.	Strong recommendation ●●●	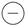	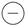
**18**	**For patients with HR+/HER2− metastatic breast cancer whose disease progresses on first-line therapy and whose tumour has an PI3K/AKT/PTEN pathway alteration (with no prior PI3K/AKT/PTEN-directed therapy)**, standard of care in the second-line setting is capivasertib + fulvestrant.	Strong recommendation ●●●	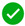	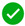
	Alpelisib + fulvestrant could be considered for patients with a confirmed *PIK3CA* mutation.	Moderate recommendation ●●		

●●●, Strong recommendation; ●●, Moderate recommendation; 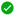, Alignment; 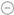, Some variation; NC, Not covered by ESMO and/or ASCO. ^1^ If ET is AI, plus OFS for premenopausal women or LHRH for men.

**Table 6:** Efficacy of oral SERDs and PROTAC in patients with *ESR1* mutations whose disease progressed on 1 to 2 prior lines of therapy, including ET + CDK4/6i.

	Intervention	Population	Median PFS	Median OS
evERA Breast Cancer [108] Phase 3 NCT05306340	Giredestrant + everolimus or SoC (exemestane/fulvestrant/tamoxifen) + everolimus	373 patients whose disease progressed or relapsed during/post-CDK4/6i + ET (100% prior CDK4/6i; 55.5% *ESR1* mutation)	9.99 vs. 5.45 HR 0.38, 95% CI 0.27–0.54, *p* < 0.0001	Interim OS NE vs. 21.03 months HR 0.62, 95% CI 0.38–1.02, *p* = 0.0566
EMERALD [107] Phase 3 NCT03778931	Elacestrant 400 mg or SoC (fulvestrant or AI)	477 postmenopausal women and men (100% prior CDK4/6i; 47.8% *ESR1* mutation)	NR HR 0.55, 95% CI 0.39–0.77, *p* = 0.0005	OS data immature Median not reported 68 events had occurred HR 0.59, 95% CI 0.36–0.96, *p* = 0.0325 (NS)
EMBER-3 [56,57] Phase 3 NCT04975308	Imlunestrant 400 mg or ET (fulvestrant or AI) or Imlunestrant 400 mg + abemaciclib 300 mg	874 women at any menopausal stage and men (59.8% prior CDK4/6i; 37.0% *ESR1* mutation)	ET: 3.8 months Imlunestrant: 5.5 months Imlunestrant + abemaciclib: 11.1 months Imlunestrant vs. SoC HR 0.62, 95% CI 0.46–0.82 *p* < 0.001 Imlunestrant + abemaciclib vs. imlunestrant HR 0.53, 95% CI 0.35–0.80	Interim OS ET: 23.1 months Imlunestrant: 34.5 months Imlunestrant + abemaciclib: NR Imlunestrant vs. SoC HR 0.60, 95% CI 0.43–0.86, *p* = 0.0043 (NS; *p*-value threshold, 4 × 10^−7^) Imlunestrant + abemaciclib vs. Imlunestrant HR 0.935, 95% CI 0.54–1.61
SERENA-2 [112] Phase 2 NCT04214288	Camizestrant 75/150 mg or Fulvestrant 500 mg	240 postmenopausal (51% prior CDK4/6i; 37% *ESR1* mutation)	Camizestrant 75: 7.2 months Camizestrant 150: 7.7 months Fulvestrant: 3.7 months Camizestrant 75 mg vs. fulvestrant HR 0.59, 90% CI 0.42–0.82, *p* = 0.017 Camizestrant 150 mg vs. fulvestrant HR 0.64, 90% CI 0.46–0.89, *p* = 0.0090	OS data immature Median reported Fulvestrant: 20.5 months Camizestrant 75/150 mg: Not reached for both groups
AcelERA BC [113] Phase 2 NCT04576455	Giredestrant 30 mg or Physician choice ET (fulvestrant or AI)	303 women at any menopausal stage and men (41.9% prior CDK4/6i; 38.8% *ESR1* mutation)	Giredestrant: 5.3 months ET: 3.5 months HR 0.60, 95% CI 0.35–1.03	OS data immature Median not reported
VERITAC-2 [114] Phase 3 NCT05654623	Vepdegestrant or Fulvestrant	624 (100% prior CDK4/6i; 43.3% *ESR1* mutation)	Vepdegestrant: 5.0 months Fulvestrant: 2.1 months HR 0.57, 95% CI 0.42–0.77, *p* = 0.0001	OS data immature Median not reported

Abbreviations: AI = aromatase inhibitor; CDK4/6i = cyclin-dependent kinase 4/6 inhibitor; CI = confidence interval; ET = endocrine therapy; HR = hazard ratio; NE, not estimable; NR = not reported; NS = not significant; OS = overall survival; PFS = progression-free survival; SoC = standard of care.

**Table 7:** REAL Alliance recommendations for third-line treatment of patients with endocrine-eligible metastatic breast cancer whose disease progresses on ET in the metastatic setting, and comparison with those from ESMO and ASCO.

Recommendations for Third-Line Treatment of Patients with Endocrine-Eligible Metastatic Breast Cancer ^1^		REAL	ESMO [9,14]	ASCO [7]
**19**	**For patients who have had two prior lines of ET (without CDK4/6i) in the metastatic setting and whose disease progresses**, a trial of CDK4/6i + ET using a different ET agent should be considered.	Moderate recommendation ●●	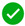	NC

●●, Moderate recommendation; 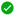, Alignment; NC, Not covered by ESMO and/or ASCO. ^1^ Plus OFS for premenopausal women or LHRH agonist for men.

**Table 8:** REAL Alliance recommendations for treatment of patients with endocrine-ineligible metastatic breast cancer, and comparison with those from ESMO and ASCO.

Recommendations for Treatment of Patients with Endocrine-Ineligible Metastatic Breast Cancer		REAL	ESMO [9,14]	ASCO [7]
**20**	**For patients with HR+/HER2− metastatic breast cancer that is ET ineligible and has progressed after prior ET + CDK4/6i in any setting**, chemotherapy, including ADC, is the standard of care.	Strong recommendation ●●●	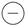	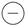
**21**	**(a) For patients with HR+/HER2-low or ultralow metastatic breast cancer that has progressed on prior ET + CDK4/6i**, T-DXd is an option as the next line of systemic therapy if no prior chemotherapy has been given, guided by shared decision-making.	Strong recommendation ●●●	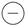	NC
	**(b) For patients with HER2-low disease who have not previously received T-DXd, but who have received at least one line of chemotherapy**, T-DXd is standard of care.	Strong recommendation ●●●	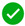	NC
**22**	**For patients with HR+/HER2− metastatic breast cancer with germline *BRCA1/2* mutations who are no longer benefiting from ET**, an oral PARP inhibitor should be considered post-ET instead of chemotherapy.	Strong recommendation ●●●	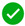	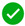
**23**	**For patients with HR+/HER2− metastatic breast cancer that has progressed and who have received ≥2 chemotherapy regimens and no prior ADC**, sacituzumab govitecan is the standard of care.	Strong recommendation ●●●	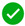	NC

●●●, Strong recommendation; 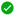, Alignment; 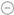, Some variation; NC, Not covered by ESMO and/or ASCO.

## Data Availability

No new data were created or analyzed in this study.

## Supplementary Materials

The following supporting information can be downloaded at: https://www.mdpi.com/article/10.3390/curroncol33020106/s1. Table S1: Voting Summary; Table S2: Summary of phase 3 clinical studies of CDK4/6i + AI for first-line treatment of HR+/HER2− metastatic breast cancer.
