# Supplementary material for: Guidance for Canadian Breast Cancer Practice: National Consensus Recommendations for the Systemic Treatment of Patients with HR+/HER2− Metastatic Breast Cancer 2025
Source: Curr Oncol. 2026 Feb 9;33(2):106. doi: 10.3390/curroncol33020106 (PMC12938948; doi:10.3390/curroncol33020106)
Supplement: Supplementary file 1 [file curroncol-33-00106-s001.zip › curroncol-4090259-supplementary.pdf]

Guidance for Canadian Breast Cancer Practice: National Consensus Recommendations for the Systemic Treatment of Patients with HR+/HER2- Metastatic Breast Cancer

Table *S1.* Voting results.

Voting results (■ Agree with statement as is, ■ Agree with statement with edits, ■ Do not agree with statement)

	Recommendation	Consensus Recommendation	Consensus was reached on:
1	If feasible, biopsy of a recurrent lesion(s) should be considered at the time of diagnosis to evaluate biomarkers (ER/PR/HER2 status) or if the clinical course is not as expected. (Moderate Recommendation)	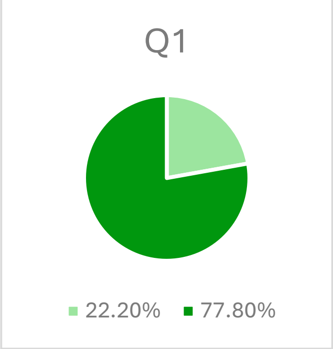<br>N=18	2nd round
2	Testing for somatic pathway alterations (e.g., *PIK3CA*, *PTEN*, *AKT*, *ESR1*) and pathogenic germline variants, (e.g., *BRCA* mutations) is standard of care when it may inform treatment decisions. Testing should be performed with appropriate methods and in a timely manner to guide treatment decisions. (Strong Recommendation)	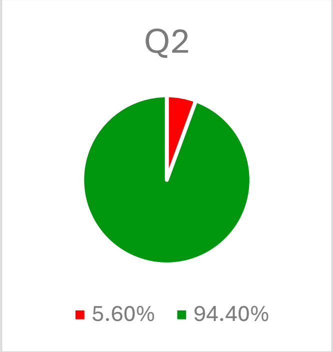<br>N=18	3rd round
3	In the management of HR+/HER2- metastatic breast cancer, it is essential to balance disease control with quality of life, allowing patients to maintain daily activities and minimize treatment-related discomfort. (Moderate Recommendation)	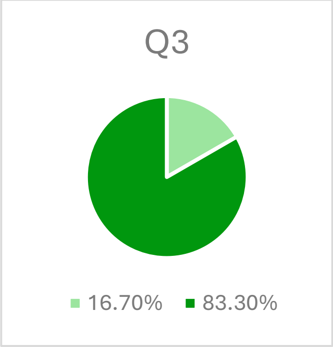<br>N=18	1st round

4	The treatment and management of male breast cancer is the same as in pre/perimenopausal women (i.e., ET + CDK4/6i + LHRH agonist). Given the rarity of male breast cancer, participation in clinical trials should be encouraged whenever possible. (Strong Recommendation)	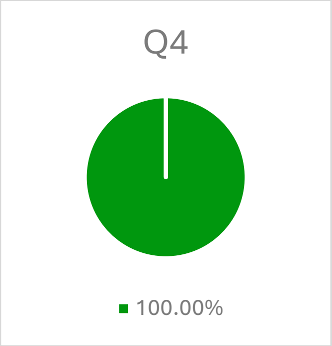 N=18	1st round
5.	For patients with HR+/HER2- metastatic breast cancer who have not received prior or recent ET, the standard of care ET backbone in the first-line metastatic setting is an AI. (Strong Recommendation)	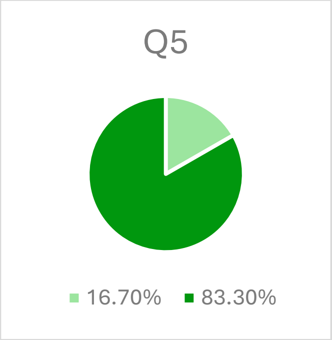 N=18	1st round
6	For patients with de novo metastatic HR+/HER2- breast cancer, or those who relapse > 12 months after completing adjuvant ET, and regardless of adjuvant treatment with CDK4/6i, the standard of care first-line treatment is CDK4/6i + AI. The preferred CDK4/6i is either ribociclib or abemaciclib; however, in the case of contraindications or intolerance, palbociclib may be considered. (Strong Recommendation)	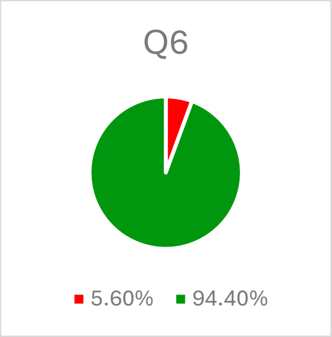 N=18	3rd round
7	For patients with HR+/HER2- breast cancer without *PIK3CA* mutation and with relapse on or within 12 months after completion of adjuvant AI, SERD is the standard of care ET backbone. (Strong Recommendation)	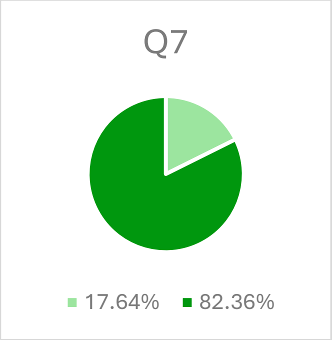 1 abstained<br>N=17	2nd round

8	For patients with HR+/HER2- breast cancer without PIK3CA mutation and with relapse on or within 12 months after completion of adjuvant AI, and without prior adjuvant CDK4/6i, the standard of care treatment is either ribociclib + fulvestrant or abemaciclib + fulvestrant. In the case of contraindications or intolerance, palbociclib + fulvestrant may be considered. (Strong Recommendation)	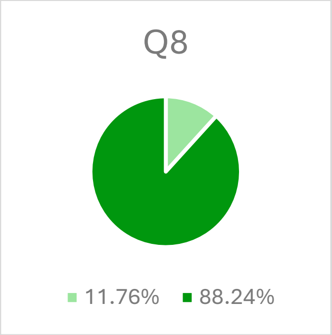 1 abstained<br>N=17	2nd round
9	For patients with HR+/HER2- breast cancer without PIK3CA mutation and with relapse on or within 12 months after completion of adjuvant AI, and with prior adjuvant CDK4/6i, rechallenge with ET + CDK4/6i should be considered, depending on the clinical situation and timing of relapse. (Moderate Recommendation)	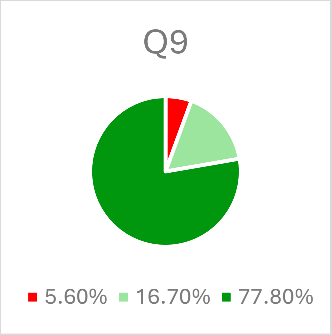 N=18	1st round
10	For patients with HR+/HER2- breast cancer with PIK3CA mutation and with relapse on or within 12 months after completion of adjuvant AI, inavolisib + palbociclib + fulvestrant is the standard of care. (Strong Recommendation)	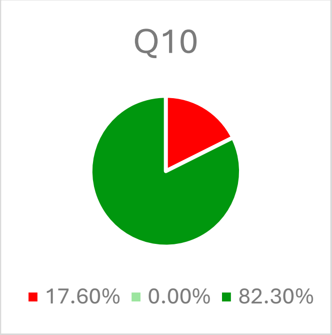 N=17<br>1 abstain	3rd round
11	For patients with HR+/HER2- metastatic breast cancer and limited life expectancy or for those who, with shared decision-making, wish not to have intensive monitoring or toxicities, ET alone is a reasonable option. (Expert Opinion)	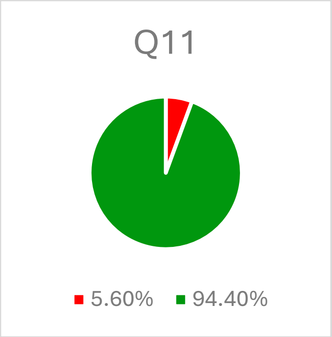 N=18	1st round

12	For eligible older patients (≥75 years of age) with HR+/HER2- metastatic breast cancer, the standard of care at standard recommended doses remains the same as that for younger patients. (Strong Recommendation)	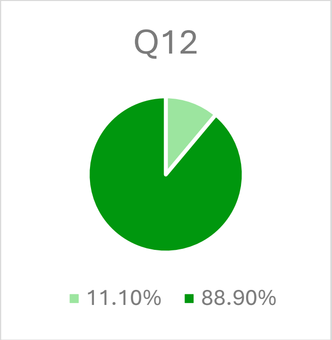<br>N=18	1st round
13	For patients with HR+/HER2- metastatic breast cancer and visceral disease and in the absence of true visceral crisis[2], ribociclib +ET or abemaciclib + ET is standard of care (instead of chemotherapy) with close monitoring for progression of disease or lack of response. Palbociclib + ET may be considered if neither ribociclib nor abemaciclib are suitable. (Strong Recommendation)	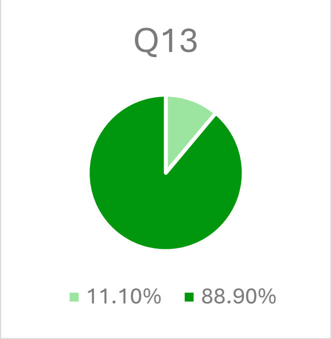<br>N=18	2nd round
14	For patients with HR+/HER2- metastatic breast cancer with bone metastases, the use of bone-modifying agents (e.g., bisphosphonates or denosumab) is standard of care to reduce and delay skeletal related adverse events. (Strong Recommendation)	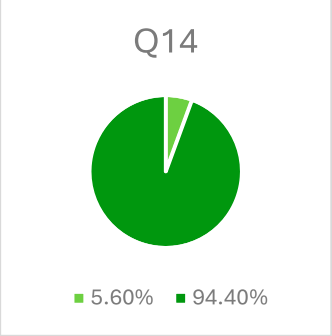<br>N=18	2nd round
15	For patients with HR+/HER2- metastatic breast cancer with central nervous system (CNS) involvement, a multidisciplinary team should be involved in providing recommendations for optimal local and systemic therapies. Currently, there is insufficient evidence to recommend any given systemic therapy alone (in the absence of local therapy) for the treatment of “active” HR+/HER2- CNS metastases. (Moderate Recommendation)	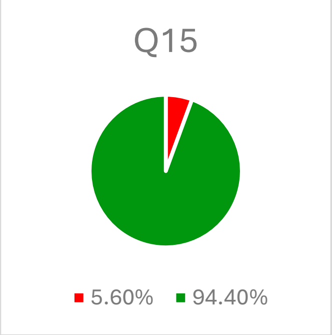<br>N=18	1st round

16	For patients with HR+/HER2- metastatic breast cancer with no targetable mutations whose disease progresses on first-line therapy of ET + CDK4/6i, switching ET + another CDK4/6i should be considered. Everolimus + ET is another consideration. (Moderate Recommendation)	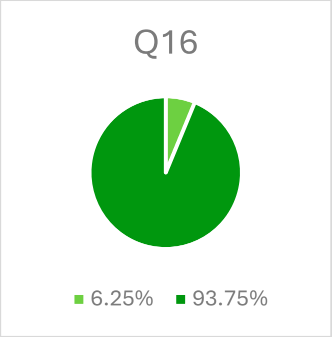<br>1 abstained<br>N=17	1st round
17	For patients with HR+/HER2- metastatic breast cancer with ESR1 mutations (without PIK3CA alterations) whose disease progresses on first-line therapy of AI + CDK4/6i, a SERD is standard of care ET, either as monotherapy or in combination with a targeted agent. (Strong Recommendation)	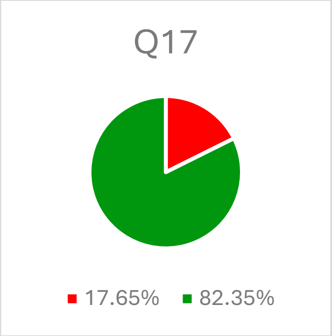<br>1 abstained<br>N=17	3rd round
18	For patients with HR+/HER2- metastatic breast cancer whose disease progresses on first-line therapy and whose tumour has a PI3K pathway alteration (with no prior PI3K-directed therapy), standard of care in the second-line setting is capivasertib + fulvestrant.<br><br>Alpelisib + fulvestrant could be considered. (Strong Recommendation)	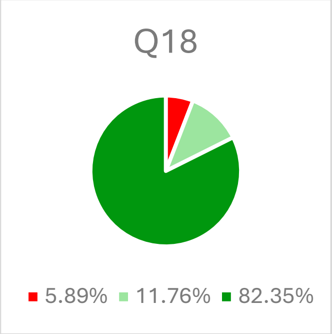<br>1 abstained<br>N=17	2nd round
19	For patients who have had two prior lines of ET (without CDK4/6i) in the metastatic setting and experience recurrence, a trial of CDK4/6i + ET using a different ET agent should be considered. (Moderate Recommendation)	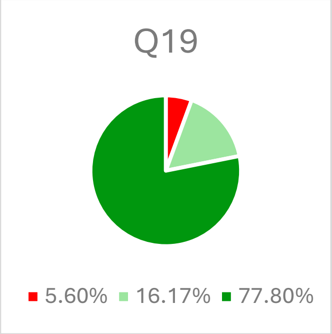<br>N=18	1st round

20	For patients with HR+/HER2- metastatic breast cancer that is ET ineligible and has progressed after prior ET + CDK4/6i in any setting, chemotherapy (including ADC) is the standard of care. (Strong Recommendation)	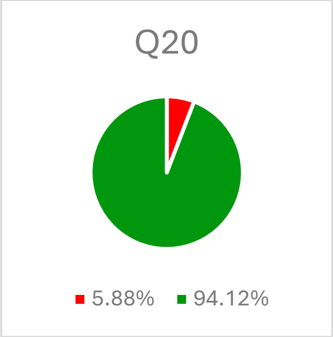<br><br>1 abstained<br>N=17	3rd round
21. a)	For patients with HR+/HER2-low or ultralow metastatic breast cancer that has progressed on prior ET + CDK4/6i, T-DXd is an option as the next line of systemic therapy if no prior chemotherapy has been given guided by shared decision-making. (Strong Recommendation)	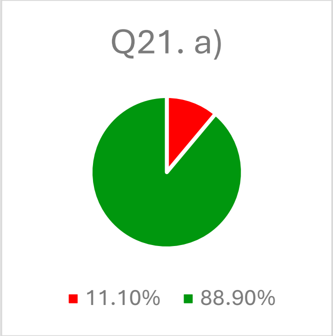<br><br>N=18	3rd round
21. b)	For patients with HER2-low disease who have not previously received T-DXd but who have received at least one line of chemotherapy, T-DXd is standard of care. (Strong Recommendation)	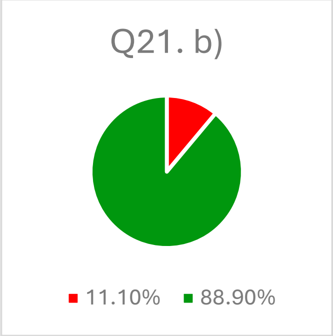<br><br>N=18	3rd round
22	For patients with HR+/HER2- metastatic breast cancer with germline BRCA1/2 mutations who are no longer benefiting from ET, an oral PARP inhibitor is standard of care as a first-, second-, or third-line treatment instead of chemotherapy. (Strong Recommendation)	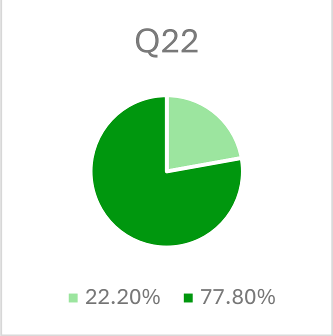<br><br>N=18	1st round

23	For patients with HR+/HER2- metastatic breast cancer that has progressed and who have received ≥2 chemotherapy regimens and no prior ADC, sacituzumab govitecan is the standard of care. (Strong Recommendation)	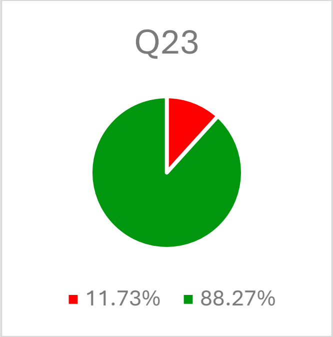 1 abstained<br>N=17	3rd round

Table S2. Summary of phase 3 clinical studies of CDK4/6i + AI for first-line treatment of HR+/HER2- metastatic breast cancer.

	**MONALEESA-7** [31,32,34]	**MONALEESA-2**[35,37]	**MONARCH-3**[38,39]	**PALOMA-2**[48,49]
**Interventions**	Ribociclib + ET vs Placebo + ET<br><br>n=495 (74%) received AI (letrozole or anastrozole)	Ribociclib + AI<br>vs Placebo + AI (letrozole)	Abemaciclib + AI<br>Vs Placebo + AI<br>(anastrozole or letrozole)	Palbociclib + AI<br>Vs Placebo + AI<br>(letrozole)
**Population**	Premenopausal<br>N=672	Postmenopausal<br>N=668	Postmenopausal<br>N=493	Postmenopausal<br>N=666
**PFS, median**	23.8 vs 13.0 months<br>• HR 0.55, 95% CI 0.44–0.69, p<0.0001<br>• Median follow-up 19.2 months	25.3 vs 16.0 months<br>• HR 0.568, 95% CI 0.457–0.704, p<0.0001<br>• Median follow-up 26.4 months	28.18 vs 14.76 months<br>• HR 0.540, 95% CI 0.481–0.698, p<0.0001<br>• Median follow-up 26.73 months	27.6 vs 14.5 months<br>• HR 0.563, 95% CI 0.461–0.687, p<0.0001<br>• Median follow-up 37.6 months
**OS, median**	NR vs 40.9 months<br>• HR 0.71; 95% CI, 0.54–0.95; p=0.00973<br>• Median follow-up 34.6 months<br><br>Exploratory<br>58.7 vs 48.0 months<br>• HR 0.76, 95% CI 0.61–0.96)<br>• Median follow-up 53.5 months	63.9 vs 51.4 months<br>• HR 0.76, 95% CI 0.63–0.93, p=0.008<br>• Median follow-up 6.6 years)	66.8 vs 53.7 months<br>• HR 0.804, 95% CI 0.637–1.015, p=0.0664<br>• Median follow-up 8.1 years	53.9 vs 51.2 months<br>• HR 0.96, 95% CI 0.78– 1.18, p=0.34<br>• Median follow-up 90.1 months
**Safety** (reported in ≥3% of patients)	Grade ≥3 AE<br>• Neutropenia (61%)<br>• Leucopenia (14%)<br>• Increased ALT (5%)<br>• Increased AST (4%)<br>• Anemia (3%)<br>• Hypertension (3%)	Grade ≥3 AE<br>• Neutropenia (62.0%)<br>• Leucopenia (21.3%)<br>• Abnormal LFTs (10.2%)<br>• Vomiting (3.6%)<br>• Fatigue (3.0%)<br>• Back pain (3.0%)	Grade ≥3 AE<br>• Neutropenia (23.8%)<br>• Diarrhea (9.5%)<br>• Anemia (7.0%)<br>• Leucopenia (8.6%)<br>• Increased ALT (6.4%)<br>• Increased AST (3.7%)	Grade ≥3 AE<br>• Neutropenia (69.1%)<br>• Leukopenia (25.2%)<br>• Infections (7.6%)*<br>• Anemia (5.8%)

Abbreviations: AE, adverse event; AI = aromatase inhibitor; ALT, alanine aminotransferase; AST, aspartate aminotransferase; CDK4/6i = cyclin-dependent kinase 4/6 inhibitor; CI = confidence interval; ET = endocrine therapy; HR = hazard ratio; LFT = liver function test; NR = not reached; OS = overall survival; PFS = progression-free survival. *1 patient receiving palbociclib + AI had a Grade 5 infection (pneumonia).
